# Multi-Scale Computational Modeling of Spatial Calcium Handling From Nanodomain to Whole-Heart: Overview and Perspectives

**DOI:** 10.3389/fphys.2022.836622

**Published:** 2022-03-09

**Authors:** Michael A. Colman, Enrique Alvarez-Lacalle, Blas Echebarria, Daisuke Sato, Henry Sutanto, Jordi Heijman

**Affiliations:** ^1^School of Biomedical Sciences, University of Leeds, Leeds, United Kingdom; ^2^Departament de Fisica, Universitat Politècnica de Catalunya-BarcelonaTech, Barcelona, Spain; ^3^Department of Pharmacology, School of Medicine, University of California, Davis, Davis, CA, United States; ^4^Department of Physiology and Pharmacology, State University of New York Downstate Health Sciences University, Brooklyn, NY, United States; ^5^Department of Cardiology, Cardiovascular Research Institute Maastricht, Maastricht University, Maastricht, Netherlands

**Keywords:** cardiac electrophysiology, calcium handling in cardiomyocytes, excitation-contraction coupling, computational modeling methods, multi-scale model

## Abstract

Regulation of intracellular calcium is a critical component of cardiac electrophysiology and excitation-contraction coupling. The calcium spark, the fundamental element of the intracellular calcium transient, is initiated in specialized nanodomains which co-locate the ryanodine receptors and L-type calcium channels. However, calcium homeostasis is ultimately regulated at the cellular scale, by the interaction of spatially separated but diffusively coupled nanodomains with other sub-cellular and surface-membrane calcium transport channels with strong non-linear interactions; and cardiac electrophysiology and arrhythmia mechanisms are ultimately tissue-scale phenomena, regulated by the interaction of a heterogeneous population of coupled myocytes. Recent advances in imaging modalities and image-analysis are enabling the super-resolution reconstruction of the structures responsible for regulating calcium homeostasis, including the internal structure of nanodomains themselves. Extrapolating functional and imaging data from the nanodomain to the whole-heart is non-trivial, yet essential for translational insight into disease mechanisms. Computational modeling has important roles to play in relating structural and functional data at the sub-cellular scale and translating data across the scales. This review covers recent methodological advances that enable image-based modeling of the single nanodomain and whole cardiomyocyte, as well as the development of multi-scale simulation approaches to integrate data from nanometer to whole-heart. Firstly, methods to overcome the computational challenges of simulating spatial calcium dynamics in the nanodomain are discussed, including image-based modeling at this scale. Then, recent whole-cell models, capable of capturing a range of different structures (such as the T-system and mitochondria) and cellular heterogeneity/variability are discussed at two different levels of discretization. Novel methods to integrate the models and data across the scales and simulate stochastic dynamics in tissue-scale models are then discussed, enabling elucidation of the mechanisms by which nanodomain remodeling underlies arrhythmia and contractile dysfunction. Perspectives on model differences and future directions are provided throughout.

## Introduction

Intracellular calcium (Ca^2+^) handling is a critical component of cardiac electrophysiology (Cheng et al., 1993; Bers, 2002; Song et al., 2015): it governs excitation-contraction coupling (ECC), is involved in multiple signaling pathways, and its impairment has been causally linked to both mechanical and electrical dysfunction of the heart (Eisner et al., 2009; Voigt et al., 2014; Clarke et al., 2015). Elucidating the fundamental mechanisms of Ca^2+^ homeostasis and the perturbations of the system in disease is therefore vital for understanding the electrophysiology of the heart and identifying better diagnostic and treatment strategies for multiple cardiovascular diseases.

One major challenge of dissecting the specific roles and contributions of the many components of intracellular Ca^2+^ handling to observed (dys)function is the complex, non-linear and multi-scale properties of the system in space and time. Spatially, Ca^2+^ sparks, the fundamental element of Ca^2+^-induced-Ca^2+^-release (CICR; see next sub-section), are controlled at the nanometer-scale in localized nanodomains referred to as dyads or couplons, yet Ca^2+^ homeostasis occurs inherently at the cellular-scale where flux balance through the membrane and subcellular transporters determines the total Ca^2+^ levels in the cell and in the Sarcoplasmic Reticulum (SR, the intracellular Ca^2+^ store). Homeostatic conditions of cardiomyocytes at the cell-level are integrated in the heterogeneous syncytium of cardiac muscle tissue where individual myocytes do not function as isolated entities. Temporally, the gating of Ca^2+^ channels occurs on sub-millisecond time-frames (Zahradníková et al., 1999); the heartbeat itself occurs on the order of a second; signaling and regulation, such as sympathetic stimulation, can occur over minutes (Heijman et al., 2011); and transcription and circadian rhythms can influence dynamics over hours or even days (Black et al., 2019; D’Souza et al., 2021). Thus, structure-function relationships from the nanometer- to the whole-heart-scales and dynamics occurring over nanoseconds to hours all contribute to the macroscopic behavior of the heartbeat.

Recent advances in experimental imaging modalities and image-analysis are enabling the super-resolution reconstruction of the structures responsible for regulating Ca^2+^ homeostasis at the nanometer scale (Baddeley et al., 2009; Crossman et al., 2011; Macquaide et al., 2015; Jayasinghe et al., 2018b; Sheard et al., 2019). Extrapolating functional and imaging data from the dyad to the whole-heart is non-trivial due to multi-scale systems interactions; it is therefore a substantial challenge using experimental techniques alone to employ integrative approaches which aim to understand how macroscopic cardiac function arises from these fundamental building blocks. Computational modeling therefore has important roles to play in helping to dissect these structure-function relationships at multiple scales and elucidate the mechanisms by which cellular phenomena translate to the whole-heart.

Over the last decade in particular there have been substantial advances in the complexity and sophistication of computational models of spatial intracellular Ca^2+^ handling. Due to the variety of independently developed models and range of contexts in which they have been applied, it can be a challenging field to get into and understand, whether one is a computational modeler wanting to use and develop these models, or an experimental researcher hoping to understand the models’ limitations and where they can be used to support one’s research. This review aims to provide an accessible entry-point for those not already familiar with these models and a useful reference for those who are. We focus on methods and approaches, in particular those for image-based and multi-scale modeling, how these differ between models, and the implications of these model differences. Applications of the models will be discussed primarily within this context; the reader is referred to previous reviews for more extensive descriptions of the role of computational modeling in elucidating the Ca^2+^-mediated mechanisms of cardiac (dys)function (Heijman et al., 2016; Maleckar et al., 2017; Vagos et al., 2018; Sutanto et al., 2020).

### Structure–Function Relationships in Ca^2+^ Homeostasis: Local Control of Ca^2+^-Induced-Ca^2+^-Release

**FIGURE 1:**
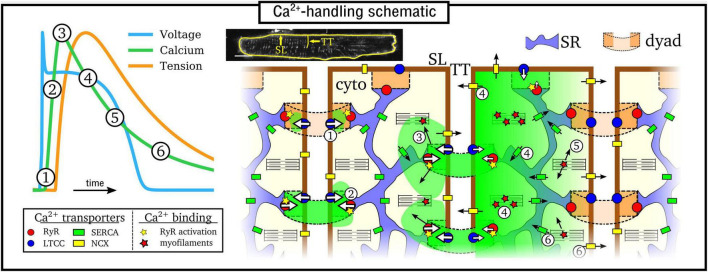
Simplified illustration of the intracellular Ca^2+^-handling cycle in ventricular myocytes. Left: illustration of an action potential (voltage), calcium transient (CaT), and developed tension, with the different stages broadly labeled on the CaT. Right: cartoon schematic of the different stages of Ca^2+^ cycling in the intracellular volume. High Ca^2+^ concentration is indicated by the green areas; SL refers to the sarcolemmal membrane and TT to a transverse tubule, illustrated on a real cell image (modified from Gadeberg et al., 2016). Numbered stages correspond to those in the main text and are illustrated at different spatial locations of the schematic for brevity – it should be clarified that all stages occur throughout the cell volume.

Excitation-contraction coupling is mediated by CICR (Cheng et al., 1993; Bers, 2002), illustrated in Figure 1: **(1)** Ca^2+^ enters the cell through the L-type Ca^2+^ channels (LTCC) during electrical excitation (the action potential, AP); **(2)** This local rise in Ca^2+^ activates the ryanodine receptors (RyRs) to trigger a large release of Ca^2+^ (triggered Ca^2+^ spark) from the SR; **(3)** Ca^2+^ diffuses throughout the myocyte, binds with the contractile apparatus, and initiates cellular contraction; **(4)** Peak contraction occurs when Ca^2+^ has diffused sufficiently throughout the cell to permit substantial binding with the contractile apparatus; Ca^2+^ influx has largely terminated at this point; intracellular Ca^2+^ is removed into the extracellular space through the sodium-Ca^2+^ exchanger (NCX) and the plasmalemmal Ca^2+^ pump, and SR-Ca^2+^ is restored through the SR-Ca^2+^ pump (SERCA); **(5)** As NCX and SERCA reduce the Ca^2+^ concentration in the intracellular volume, myofilaments release Ca^2+^ from their binding sites and cellular relaxation occurs; **(6)** NCX and SERCA continue to act to restore resting Ca^2+^ levels, ready for the next cycle.

The rise and decay of Ca^2+^ in the intracellular volume is referred to as the intracellular Ca^2+^ transient (CaT), and broadly follows the AP (Figure 1). Rather than being a whole-cell, homogeneous event, the CaT is the summation of many thousands of locally controlled Ca^2+^ sparks, conferred in the specialized nanodomains called dyads or couplons which co-localize the RyRs on the SR membrane with the LTCCs on the sarcolemma membrane. Cardiomyocytes therefore feature an intracellular structure which facilitates whole-cell contraction mediated by this local control of CICR: the SR forms a cell-wide network coupling the spatially distributed dyads throughout the intracellular volume; the surface sarcolemma (SL) membrane contains multiple invaginations into the cell interior, consisting of the transverse-tubule (TT) and axial-tubule (AT) system (T-system), harboring LTCCs, NCX, and other ion channels (Dibb et al., 2022) which enables dyads to be formed throughout the cellular volume. The reader is referred to the previous works and reviews by Cannell and Kong (2012, 2017) and Laver et al. (2013) for discussion about the importance of local control to explain the properties of CICR in the heart.

Although the Ca^2+^-handling system is conceptually similar and involves the same machinery in all regions of the heart, there are functional and structural differences between myocytes from the pacemaker regions, cardiac conduction system, atria and ventricles that are important for normal physiology as well as the genesis of cardiac arrhythmias (Sutanto et al., 2020). For example, atrial myocytes do not have as robust and dense a T-system as ventricular myocytes (Richards et al., 2011), featuring more orphaned RyR clusters (those without associated LTCCs); in the pacemaker cells of the sinoatrial and atrioventricular nodes, the Ca^2+^ handling system forms the Ca^2+^-clock which is involved in the generation of APs and does not function primarily to initiate cellular contraction (Maltsev and Lakatta, 2013; Yaniv et al., 2015; Maltsev et al., 2017). Discussing models of pacemaker and conduction system myocytes is beyond the scope of the current review, which will focus on the working myocardium of the ventricles and atria.

### Multi-Scale Dynamics of Ca^2+^ Handling

**FIGURE 2:**
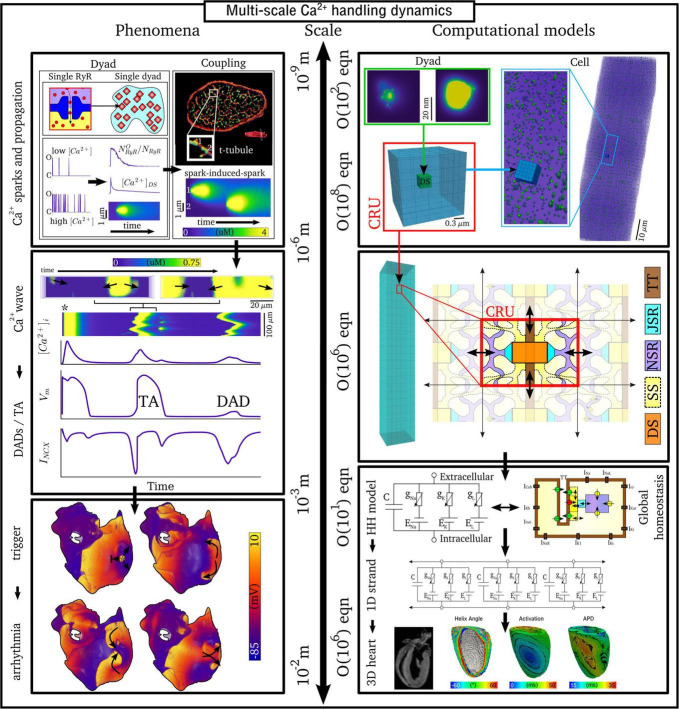
Illustration of the scales involved in cardiac Ca^2+^-handling electrophysiology. Left, upper: illustration of a single RyR and the distribution of RyRs in a dyad, RyR channel function at low and high local Ca^2+^, and propagation of a Ca^2+^-spark between dyads. Cell-structure image re-rendered using data from Jayasinghe et al. (2018a). Left, middle: Illustration of an intracellular Ca^2+^-wave and the impact of the spontaneous Ca^2+^-transient on NCX and the action potential (AP), demonstrating both a delayed-after-depolarization (DAD) and triggered AP (TA). Left, lower: illustration of a focal excitation in tissue which degenerates into a re-entrant excitation pattern. Simulation data from Colman et al. (2013). Right, upper: spatial model of a single dyad (model as presented in Sheard et al., 2019) and illustration of a sub-micron full-cell model with dyads distributed therein. Right, middle: illustration of a coarse-grained 3D cell model with different intracellular and SR compartments labeled. Right, lower: illustration of standard, non-spatial Hodgkin-Huxley electric circuit models of cardiac electrophysiology, their coupling in a 1D strand, and implementation in 3D whole-heart models based on imaging data, reproduced using data from Benson et al. (2011).

The inherently multi-scale nature of cardiac Ca^2+^-handling is perhaps best illustrated by considering the mechanisms of spontaneous Ca^2+^ release events (SCRE) and their involvement in proarrhythmogenic premature focal excitations (Figure 2).

One consequence of local control is that each dyad contains only small numbers of channels, with typical values thought to be 5–15 LTCCs and 5–200 RyRs (Baddeley et al., 2009; Jayasinghe et al., 2018a,b), located within a very small volume of order < 10^–3^ μm^3^ (Scriven et al., 2013). Stochastic oscillations of single RyR channels can result in small-scale intracellular Ca^2+^-release (Ca^2+^ quarks), potentially inducing the nanodomain-wide event of a spontaneous Ca^2+^ spark by recruiting further RyRs within the dyad to sustain a release flux. The specific mechanism for this recruitment is either a large flux of Ca^2+^ through the pore of a channel that stochastically opens, which raises the local Ca^2+^ sufficiently to trigger the opening of adjacent RyRs, or a cascade of events where a single channel opening increases the probability of opening of a second round of receptors, which triggers more rounds of receptors opening (Asfaw et al., 2013). Irrespective of the specific mechanism, once a reasonably large spark is originated, spatial-diffuse coupling provides a substrate for the propagation of Ca^2+^-sparks throughout the cell as a spark-induced-spark mediated Ca^2+^-wave (Figure 2).

These SCRE are potentially pro-arrhythmic cellular phenomena: Ca^2+^ release can activate NCX which results in a transient inward current when Ca^2+^ is extruded, depolarizing the cell membrane potential as a delayed-after-depolarization (DAD) or, if of sufficient magnitude, a full triggered AP (TA; Figure 2). Multiple cells must undergo some degree of synchronization of these TA in order for them to overcome electrotonic load and manifest in tissue as a focal excitation (Xie et al., 2010; Campos et al., 2015; Liu et al., 2015; Colman, 2019). Similar considerations apply for many sub-cellular Ca^2+^ handling phenomena, from rate-dependence to arrhythmogenic CaT alternans; the fundamental pumping function itself ultimately depends on these multi-scale interactions and can thusly be potentially perturbed by random, stochastic oscillations at the nanometer-scale. Ca^2+^-dependent regulation of the membrane potential is one of the key factors in understanding arrhythmogenesis. Elucidating these mechanisms is, alongside ECC and contractile function, a primary motivation for the development of biophysically detailed models of intracellular Ca^2+^ handling. For a comprehensive overview of the multi-scale implications of Ca^2+^ handling in normal and abnormal cardiac function, the reader is referred to, for example, the reviews of Eisner et al. (2009, 2017).

### Multi-Scale Computational Models of Spatial Ca^2+^ Handling

Due to the importance of local control, common-pool models of the cell – that is, those which treat the intracellular space as single homogenized volumes – fail to properly capture the underlying mechanisms and dynamics of Ca^2+^ handling. For example, Sato et al. (2013) demonstrated that stochasticity in Ca^2+^ cycling is necessary to explain the emergence of discordant alternans, which cannot be reproduced with deterministic, common-pool cell models. Models which explicitly account for the spatial nature of the cardiomyocyte as well as stochastic dynamics of the RyRs and LTCCs are therefore much better suited to detailed analyses of Ca^2+^-handling phenomena. However, these models are also computationally more intensive than common-pool models, by a factor of >10^5^, and less suitable for tissue-scale and especially whole-heart simulations. Therefore, different models need to be considered at different spatial scales (Figure 2).

This review will discuss models of spatial Ca^2+^ handling at the multiple scales of the single nanodomain, the cardiomyocyte, and the whole-heart. Models describing the kinetics of the RyRs will first be discussed, followed by spatial models of the single nanodomain. Approaches to whole-cell modeling will then be discussed, with a particular focus on mechanisms of spatial Ca^2+^ coupling. This discussion will then be expanded to approaches for modeling variable and heterogeneous sub-cellular structure and the integration of experimental imaging data. Finally, approaches to develop simplified, computationally efficient models which still capture important features of spatial and stochastic Ca^2+^ handling will be discussed, in both the context of providing generalizable mechanistic explanations and for performing tissue-scale simulations of many thousands or millions of coupled cells. Overall clarity is prioritized over providing substantial details of all available models and investigations; thus, this review should not be considered exhaustive. The reader is also referred to the extensive overview of multi-scale mathematical and computational modeling methods presented in Qu et al. (2014).

## Modeling Ca^2+^-Induced-Ca^2+^-Release: Descriptions of Ryanodine Receptors’ Kinetics

There are multiple descriptions of RyR kinetics which have been used in computational modeling. The simplest form are two-state models, which have only a closed/inactivated state and an open/activated state. Most frequently used are four-state models, which have more details of refractoriness and inactivation (which may or may not physiologically occur, discussed in the later sub-section “Perspectives on model differences”) and can simulate different potential mechanisms of these behaviors. The model structure of the RyR is directly related to the type of behavior that is considered to be behind the appearance of local sparks. In two-state models, refractoriness is not considered relevant in the dynamics of initiation and termination of sparks, whereas refractoriness and more complex gating, that may play critically important roles in the regulation of spark dynamics, can be included in four-state and other models. Previous studies and reviews have compared fundamentally different models of the RyR regarding their ability to reproduce different features of the physiology and/or assess the ability of different proposed mechanisms to explain these features. For example, Stern et al. (1999) evaluated different Markov-chain model constructions for reproducing CICR, and Cannell and Kong (2012, 2017) assessed different mechanisms of Ca^2+^-spark termination.

This section aims to provide a clear indication of the models used in various studies by different groups and explain what the major features of these models’ differences are, in order to guide the reader through the many studies. The focus is primarily on those models which are utilized in whole-cell simulations, rather than those designed specifically to evaluate RyR function in isolation or in bilayers, such as Zahradníková et al. (1999).

### Model Structure

**FIGURE 3:**
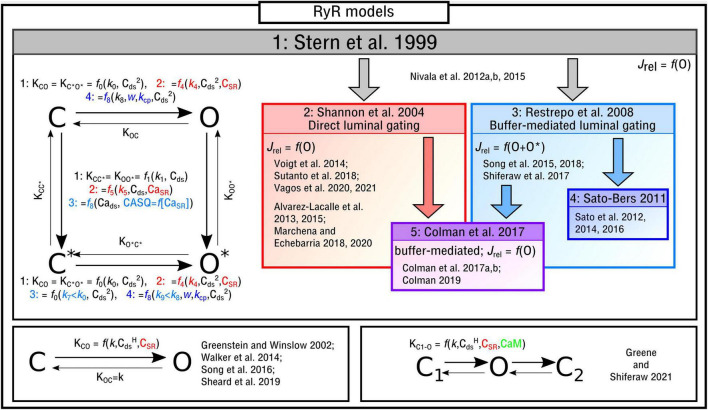
Illustration of RyR Markov-chain models. The four-state, two-state and three-state models are illustrated with the different features highlighted and a selection of publications in which they are used listed. Block arrows indicate model inheritance.

The four-state Markov chain RyR model (Figure 3) which forms the basis for the majority of modeling studies, originally presented in Stern et al. (1999), is governed by the following state-equations:


(1) $$\frac{dC}{dt}=O\cdot K_{O-C}+C^{{}^{*}}\cdot K_{C^{{}^{*}}-C}-C\cdot \left(K_{C-O}+K_{C-C^{{}^{*}}}\right)$$

(2) $$\frac{dO}{dt}=C\cdot K_{C-O}+O^{{}^{*}}\cdot K_{O^{{}^{*}}-O}-O\cdot \left(K_{O-C}+K_{O-O^{{}^{*}}}\right)$$

(3) $$\frac{dC^{{}^{*}}}{dt}=O^{{}^{*}}\cdot K_{O^{{}^{*}}-C^{{}^{*}}}+C\cdot K_{C-C^{{}^{*}}}-C^{{}^{*}}\cdot \left(K_{C^{{}^{*}}-O^{{}^{*}}}+K_{C^{{}^{*}}-C}\right)$$

And by definition/conservation:


(4) $$O^{{}^{*}}=1-\left(C+O+C^{{}^{*}}\right)$$

Where O, O*, C, and C* represent the four states of the model and correspond to different physical conditions dependent on the specific model implemented (e.g., active; refractory; inactivated; buffer binding state). The release flux, *J*_*rel*_, is generally given by:


(5) $$J_{rel}=J_{rel}^{\max}\cdot O\cdot \left({\left[Ca^{2+}\right]}_{SR}-{\left[Ca^{2+}\right]}_{i}\right)$$

Where *J*_*rel*_*^max^* is the maximal flux rate and the subscripts *SR* and *i* denote the SR and intracellular Ca^2+^ concentrations. The models have the following symmetries (some of which are broken in further model developments):


(6) $$K_{C-O}=K_{C^{{}^{*}}-O^{{}^{*}}}$$

(7) $$K_{O-C}=K_{O^{{}^{*}}-C^{{}^{*}}}$$

(8) $$K_{C-C^{{}^{*}}}=K_{O-O^{{}^{*}}}$$

(9) $$K_{C^{{}^{*}}-C}=K_{O^{{}^{*}}-O}$$

If all of these symmetries are preserved, the description becomes equivalent to a Hodgkin-Huxley model with two independent gating variables.

The functional form of these transition rates, including the variables that they depend on and which states correspond to release flux, differ between the different implementations. In the original study by Stern et al. (1999), and henceforth referred to as the “Stern-like” models, one single state (O) corresponds to the release flux [equation (5)]; transitions from the closed-to-open states, which are symmetric for C-O and C*–O*, are dependent on the square of local cytosolic Ca^2+^ (“Ca_*i*_” from herein for brevity and to avoid confusion with notation of powers); transitions from the top-to-bottom, also symmetric, are linearly dependent on the local Ca_*i*_:


(10) $$K_{C-O}=K_{C^{{}^{*}}-O^{{}^{*}}}=f_{0}\left(Ca_{i}^{2}\right)=k_{0}Ca_{i}^{2}$$

(11) $$K_{C-C^{{}^{*}}}=K_{O-O^{{}^{*}}}=f_{1}\left(Ca_{i}\right)=k_{1}Ca_{i}$$

(12) $$K_{O-C}=K_{O^{{}^{*}}-C^{{}^{*}}}=k_{2}$$

(13) $$K_{C^{{}^{*}}-C}=K_{O^{{}^{*}}-O}=k_{3}$$

Where *k*_0–3_ are constants. This model is directly used in, for example, Nivala et al. (2012a,b, 2015).

Shannon et al. (2004) updated this formulation to introduce the SR-Ca^2+^ (Ca_SR_ for brevity) as a gating variable, which will be referred to here as “direct luminal gating.” The transition rates from the closed-to-open states are now functions of Ca_SR_ as well as Ca_*i*_^2^, and the transitions from active to inactive (top-to-bottom) are dependent on both Ca_SR_ and Ca_i_:


(14) $$K_{C-O}=K_{C^{{}^{*}}-O^{{}^{*}}}=f_{4}\left(Ca_{i}^{2},Ca_{SR}\right)=\left(\frac{k_{4}}{k_{CaSR}}\right)Ca_{i}^{2}$$

(15) $$K_{C-C^{{}^{*}}}=K_{O-O^{{}^{*}}}=f_{5}\left(Ca_{i},Ca_{SR}\right)=k_{5}k_{CaSR}Ca_{i}$$

Where


(16) $$k_{CaSR}=SR^{\max}-\left(SR^{\max}-SR^{\min}\right).{\left[1+{\left(\frac{EC_{50}^{SR}}{Ca_{SR}}\right)}^{H}\right]}^{-1}$$

This formulation is one of the most-commonly used (Alvarez-Lacalle et al., 2013, 2015; Voigt et al., 2014; Marchena and Echebarria, 2018, 2020; Sutanto et al., 2018; Vagos et al., 2020, 2021).

Whereas the above model introduced direct luminal gating, many models include “buffer-mediated luminal gating,” wherein the Ca_SR_ influences gating of the RyRs not directly but rather mediated through the SR-Ca^2+^ buffer calsequestrin (CASQ), as introduced by Restrepo et al. (2008) and similar to that of Gaur and Rudy (2011). The closed-to-open transition rates have the same form as the original Stern formulation and the top-to-bottom rates are now dependent on CASQ. Two major differences are: (1) There are now two open states (O and O* in the illustration) with the formulation for *J*_rel_ [equation (5)] updated accordingly [equation (21)]; (2) The constant for the open transition rate for the lower portion of the model (which now corresponds to CASQ-bound) is smaller than that of the top, breaking one of the symmetries in the previous models [equation (6) is no longer true]:


(17) $$K_{C-O}=f_{6}\left(Ca^{2}\right)=k_{6}Ca_{i}^{2}$$

(18) $$K_{C^{{}^{*}}-O^{{}^{*}}}=f_{7}\left(Ca^{2}\right)=k_{7}Ca_{i}^{2}$$

Where


(19) $$k_{6}>k_{7}$$

And CASQ determines the unbound-bound transition rates:


(20) $$K_{C-C^{{}^{*}}}=K_{O-O^{{}^{*}}}=f_{8}\left(Ca_{i},CASQ\right)$$

The release flux is now given by:


(21) $$J_{rel}=J_{rel}^{\max}\cdot \left(O+O^{{}^{*}}\right).\left(Ca_{SR}-Ca_{i}\right)$$

The reader is referred to the work of Restrepo et al. (2008) for details of the CASQ buffering and gating equations [equation (20)]. Models which use this formulation include Song et al. (2015, 2017, 2018) and Shiferaw et al. (2017).

In Sato and Bers (2011) and subsequent studies (Sato et al., 2012, 2014, 2016), this model was updated in order to reduce the number of RyRs open during a Ca^2+^ spark:


(22) $$K_{C-O}=f_{8}\left(Ca_{i}^{2}\right)=\frac{k_{8}Ca_{i}^{2}}{k_{cp}^{2}+Ca_{i}^{2}}+w$$

(23) $$K_{C^{{}^{*}}-O^{{}^{*}}}=f_{9}\left(Ca_{i}^{2}\right)=\frac{k_{9}Ca_{i}^{2}}{k_{cp}^{2}+Ca_{i}^{2}}+w$$

Where *k*_cp_ and *w* are further constants. The majority of the models presented in the field – especially those of whole-cells – implement an RyR model which falls into one of these three broad categories. It is worth noting that the relatively simple functional forms of the transition rates given for the different models above may be modified in studies which aim to fit to experimental data describing RyR open probability, e.g., Voigt et al. (2014), Sutanto et al. (2018), and Vagos et al. (2020); these studies contain further parameters and more complex functions, but their Ca_i_ and Ca_SR_ dependence is still captured in the general forms of the model given above. Further alternatives and updates exist of these baseline models. In Colman et al. (2016, 2017a,2017b) and Colman (2019), a functionally motivated hybrid was developed wherein only one state corresponds to the open condition but the luminal dependence is buffer-mediated rather than direct.

One feature of the models, which is independent of the fundamental model structure but nonetheless important for model behavior, is the Hill coefficient, H, to which Ca_i_ is raised by for the closed-to-open (left-to-right) transition rates. Many of the models use a simple coefficient of *H* = 2. However, single-channel and single-nanodomain studies (Sobie et al., 2005; Cannell et al., 2013) indicate that H is species-dependent and varies in the range 2–2.8 which may be implemented in some studies.

### Alternative Models of the Ryanodine Receptors

There are alternative formulations to describe RyR kinetics which either: (1) are not of the form of a four-state model or (2) introduce further environmental variables to control gating. In Song et al. (2016), a reduced two-state approximation of the four-state RyR model was introduced in a study focused on elucidating long-lasting Ca^2+^ sparks. A reduced, or minimal, two-state model (Figure 3) was used in studies of single nanodomains (Greenstein and Winslow, 2002; Walker et al., 2014; Sheard et al., 2019). In Greene and Shiferaw (2021) a three-state RyR model (Figure 3) was implemented which does not correspond directly to a reduction of the four-state model. The model included a second closed state after the open state, which was introduced to reproduce “flicker” based on Mukherjee et al. (2012), as well as containing regulation of the RyRs by Calmodulin (CaM). CaM was also included in the deterministic model presented in Wei et al. (2021).

There is also the question of whether allosteric interactions/cooperativity play a role in RyR gating, with Marx et al. (2001) demonstrating that the regulatory subunit FK506-binding protein could functionally couple neighboring RyRs to underlie coordinated gating. These interactions are included in many works (Stern et al., 1999; Sobie et al., 2002; Chen et al., 2009; Greene and Shiferaw, 2021) and have been proposed as one explanation for self-termination of the Ca^2+^ spark, as discussed in Cannell and Kong (2017), although as argued in that review, unlikely to be a major contributor to this phenomenon.

### Numerical Solutions to the Ryanodine Receptors Model

In common-pool models of the cardiomyocyte, the solutions to the RyR/LTCC models are typically numerically approximated using deterministic algorithms such as the forward-Euler method. These numerical solutions correspond to tracking only the average state of the system, i.e., the proportion of open RyRs/LTCCs across the whole-cell; information on the state of individual channels or channel clusters is not preserved in such an approximation. One motivation for the development of detailed spatial models of the dyad or cardiomyocyte is to capture the stochastic (random) nature of individual RyRs/LTCCs as well as their local control, due to the relevance of both of these features for both CICR and more complex emergent dynamics such as Ca^2+^-waves. Thus, deterministic solutions are no longer suitable. Instead, stochastic algorithms that explicitly account for randomness and track individual channels are required.

The most straight-forward method is to implement the Monte-Carlo approach: the state of each individual channel is tracked directly, and state-transitions are determined based on random numbers and the probability of transition. For example, for a two-state RyR model corresponding to only closed (C) and open (O) states, the algorithm at each time-step (Δ*t*) might look like:

Loop over all RyRs:
RAND = generate random number between 0 and 1
IF state is equal to C:
IF RAND < *K*_C–O_ × Δ*t*: state becomes O
ELSE state remains C
ELSE IF state is equal to O:
IF RAND < *K*_O–C_ × Δ*t*: state becomes C
ELSE state remains O

The release flux is then given by the sum of the open channels in each dyad. This approach is ideally suited to cases where each individual channel is required to be tracked, for example in spatial models of the single nanodomain (Mesa et al., 2021). However, in larger models, e.g., of the whole-cell, this then requires the state of ∼50 RyRs + ∼15 LTCCs per dyad × ∼20,000 dyads to be tracked individually, with random numbers generated to determine state transitions for each one, which can be computationally intensive. There are more sophisticated approaches that can capture these same dynamics but at a reduced computational cost, such as a modified Gillespie’s algorithm (Gillespie, 1976; Rathinam et al., 2003; Nivala et al., 2012a; Song et al., 2019), or the Fokker-Planck or Langevin equations (Herzel, 1991; Wang et al., 2015), which can be briefly summarized as the addition of noise to a deterministic solution. Further approaches to capture the stochastic nature of RyR dynamics at a reduced computational cost, suitable for large-scale tissue simulations, are described in the final section of this review: “Simplified, minimal and tissue models.”

### Perspectives on Model Differences

The disparity between the gating mechanisms of the different models could have important implications for model dynamics and thus mechanistic conclusions drawn from these simulations. Due to inter-model differences in the setup of the whole-cell (see section “Spatial models of the whole cardiomyocyte”), it is not necessarily trivial to directly evaluate RyR function, as dynamics are intricately linked with other model parameters such as dyadic cleft volume, LTCC formulation/magnitude, local Ca^2+^ buffering, and spatial Ca^2+^ coupling; an RyR model often cannot simply be “dropped in” or “swapped out” in a whole-cell model. One major difference in model function is the typical number (or proportion) of RyRs that open in a given cluster during a triggered Ca^2+^ spark, as directly addressed in the reformulation presented in Sato and Bers (2011). A similar model structure (e.g., the same four states and functional dependence on local Ca^2+^ concentrations) but with different parameters governing the transition rates and maximal Ca^2+^-flux rate may lead to very different outcomes in adaptive function regarding Ca^2+^-spark dynamics and homeostasis.

Another difference is the description of RyR inactivation and refractoriness, corresponding to the multiple approaches discussed above (e.g., no inactivation, luminal gating, buffer-mediated gating). One reason for the disparities between the model structures is the current debate regarding the fundamental physiological relevance of RyR inactivation (Cannell and Kong, 2017), where there is no strong evidence that significant inactivation occurs under physiological conditions. However, there are strong indications of interactions between CaM binding sites and opening properties of the RyR that may be relevant for mathematically equivalent states to inactivation: Wei et al. (2021), for example, demonstrate that inactivation may be mediated by CaM-Ca^2+^ binding to the RyR and that this plays a significant role in CaT alternans. An important implication of the model choice to either include or omit significant RyR inactivation is the degree to which the junctional SR depletes during a triggered Ca^2+^-spark: models which do not include RyR inactivation (e.g., Hake et al., 2012; Cannell et al., 2013) exhibit substantially greater depletion of local SR-Ca^2+^ concentrations than those which do include inactivation (e.g., Restrepo et al., 2008; Colman et al., 2017b; Sutanto et al., 2018). This further highlights the challenge of evaluating RyR models under the same environmental cell conditions: the extent of junctional SR depletion will have large implications on homeostasis when combined with the specific formulations and parameterization of the Ca^2+^ buffers, SERCA and NCX, which primarily control the balance of SR-Ca^2+^-refilling and cellular Ca^2+^ efflux. Recent studies have also highlighted the direct importance of SERCA function for Ca^2+^ homeostasis and the dynamics of Ca^2+^ sparks (Hake et al., 2012; Sato et al., 2016, 2021; Holmes et al., 2021).

A further factor, which has recently been included in Berti et al. (2017), is the role of other ions such as K^+^, Mg^2+^, Cl^–^, and the counter-ion fluxes they facilitate during SR-Ca^2+^ release which help to maintain the trans-SR membrane driving force, especially at rapid pacing rates. It is possible that these dynamics could also influence RyR refractoriness by modulating this driving force.

Considerations of further complexity, such as the inclusion/omission of regulation by CaM (or other potential RyR and Ca^2+^ signaling modulators) can be directly motivated by the aim of the specific study; it is generally the perspective that additional complexity should only be included where specifically required, in order to reduce the influence of the propagation of unknown errors. However, it could also be argued that a non-linear, multi-scale complex system such as this presents the possibility for unpredictable emergent phenomena, which may depend on the interaction of factors such as CaM with other variables. In this case, it can be argued that one should aim to include as many (rigorously derived) components of the system as is feasible. Perspectives on this are ultimately philosophical and it would be unwise to disregard either argument.

Whereas the original study of Stern et al. (1999) compared multiple formulations of the RyR, this necessarily did not include the more recent updates described above (Shannon et al., 2004; Restrepo et al., 2008; Sato and Bers, 2011; Song et al., 2016; Greene and Shiferaw, 2021). Thus, a comprehensive benchmarking study which determines the implications of these model differences in relation to multiple dynamic Ca^2+^-handling-mediated phenomena would be hugely beneficial. Previous reviews and studies, such as Cannell et al. (2013), have performed this in specific contexts such as in the evaluation of the mechanism of termination of Ca^2+^ sparks, but a more holistic benchmarking study which considers RyR function in the context of multiple relevant factors simultaneously (such as CICR, Ca^2+^ spark termination, spatial Ca^2+^ coupling, spontaneous Ca^2+^ spark dynamics, responses to changes in pacing rate and cell environment, reproduction of alternans or after-depolarization) has yet to be performed. Such a study, requiring the whole-cell models described later in this review, could be hugely valuable in understanding the features of the different models and revealing fundamental insight into the physiology of cardiac Ca^2+^ handling.

## Spatial Models of the Single Dyad/Nanodomain

Only recently have experimental imaging techniques been able to resolve individual RyRs (Protasi et al., 1998; Baddeley et al., 2009; Crossman et al., 2011; Macquaide et al., 2015; Jayasinghe et al., 2018b; Sheard et al., 2019), enabling the structure of dyads (including number and arrangement of RyRs) to be described in detail. However, it is still challenging to correlate local Ca^2+^ concentration to RyR activity and quantify the specific fractional opening of RyRs during a typical spark in imaging data, and optical methods are limited to the close proximity of the cell surface. Thus, computational models of the single, spatially distributed dyad are useful to understand the mechanisms of Ca^2+^ sparks and their dependence on RyR number and arrangement.

**FIGURE 4:**
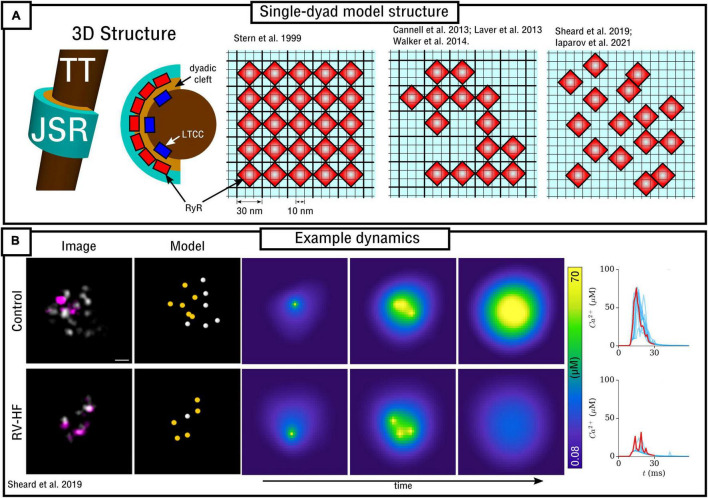
Spatial models of the dyad. **(A)** Illustration of model structure (left) and different approaches to RyR arrangement (right), ranging from regular lattice through irregular lattice to free-placement. **(B)** Illustration of a triggered Ca^2+^ spark occurring in two different RyR arrangements, corresponding to control and right-ventricular heart failure (RV-HF) directly reconstructed from imaging data. Scale bar corresponds to 50 nm. Reproduced with data from Sheard et al. (2019).

In the original paper by Stern et al. (1999), which evaluated multiple RyR Markov-chain structures including the four-state model described in detail in the previous section, dynamics were evaluated using a spatial model of the nanodomain. The 2D model was discretized at a resolution of 10 nm, with RyRs being arranged in a regular lattice with spacing 30 nm (i.e., each RyR of size 30 nm occupies a 3 × 3 grid at *dx* = 10 nm; Figure 4A). Dynamics were evaluated using different numbers of RyRs in the dyad. Whereas Ca^2+^ could diffuse within the dyadic space, the rapid equilibrium approximation was generally implemented in order to improve computational efficiency, a far more pressing constraint in 1999 than presently.

In Louch et al. (2010) a 3D cylinder model of the dyad was employed to assess the causes of dyssynchronous Ca^2+^ release in heart failure. The model was combined with AP recordings from control and heart failure myocytes. They observed that whereas AP prolongation reduces the driving force for Ca^2+^ entry through the LTCCs, this is balanced by the increase in RyR sensitivity which results from steady-state increased SR-Ca^2+^. Thus, they conclude that other factors (such as T-system disruption, see section “Modelling variability in sub-cellular structure and function”) underlie the loss of spatial synchrony in the CaT.

A detailed 3D model of multiple structures surrounding a dyad, reconstructed from electron tomograms of a mouse ventricular myocyte, was presented by Hake et al. (2012). Whereas not focusing on RyR distribution, the model included a substantial level of detail in the structures of the junctional SR (which was split into three parts of the back, release, and rim locations) and local T-tubules. The model implemented a phenomenological description of RyR gating as well as local buffers (including CASQ) and fluxes (including SERCA and NCX), and revealed the substantial gradients in local Ca^2+^ in the cytosol which emphasized the importance of the location of SERCA and its role in facilitating long-lasting Ca^2+^ sparks.

Walker et al. (2014) developed a 3D model of the dyad, junctional SR and TT based on the previous work of Sobie et al. (2002) and Williams et al. (2011). Whereas Ca^2+^ could diffuse in 3D, the RyRs were arranged on a 2D pancake (Figure 4A). Similar to the Stern et al. (1999) model, the spatial resolution was ∼10 nm with minimum RyR-spacing of ∼30 nm; RyRs were arranged on this regular lattice but now in irregular patterns. This study demonstrated the importance of specific RyR arrangement – not just total number – on Ca^2+^ spark dynamics and therefore highlighted the importance of Ca^2+^ diffusion within the dyad.

A similar model was also presented in Cannell et al. (2013) and Laver et al. (2013), which included a network SR with SERCA and its associated intracellular uptake flux. The RyR model was specifically fit to Ca^2+^ concentrations corresponding to CICR and thus not suitable for quiescent/spontaneous spark analysis. These models supported the mechanism of induction-decay for Ca^2+^-spark termination, described mathematically by Hinch (2004) and referring to the mechanism by which reduced release flux as the SR depletes results in increased closed-times of adjacent RyRs and thus an increase in the probability of spontaneous spark termination, as a direct consequence of the steep relationship between local Ca^2+^ and the closed-to-open transition rate for the RyRs. This work also demonstrated that other more complex mechanisms (such as inactivation of the RyRs, discussed earlier) were not necessary to explain experimental observations, although they do state that they likely still play a role facilitating multiple points of control.

In Sheard et al. (2019) a 2D model was presented based directly on super-resolution imaging data. The model was also discretized at a resolution of 10 nm with minimum RyR-spacing of 30 nm. In this case, RyR positions were not arranged in a lattice, but were freely placed, directly determined by experimental images of multiple dyads in both control and right-ventricular heart failure (RV-HF) conditions (Figure 4). This model also included differentiation between non-phosphorylated and phosphorylated RyRs (modeled as a simple increase in sensitivity to Ca^2+^), and results support the conclusions of both Cannell et al. (2013) and Walker et al. (2014) regarding the importance of both number and specific arrangement of RyRs for triggered Ca^2+^ spark dynamics and fidelity. Mesa et al. (2021) also investigated the functional impact of selected phosphorylated RyRs within a cluster, indicating that they can play a compensatory role in recovering healthy spark dynamics which had been lost through cluster disruption. Iaparov et al. (2021) developed a 2D model which included further possibilities for RyR arrangement and spacing (including both approaches outlined above), number, and dyad extent. They found that Ca^2+^ spark occurrence varied with the spatial arrangement, but did not consistently correlate with total RyR number, the magnitude of Ca^2+^ current or the surface density. This model included allosteric interactions and the authors found RyR coupling strength to be a major factor underlying sparks.

### Challenges and Future Directions; Importance of Spatial Ca^2+^ Coupling

As the experimental structural data improves in resolution and quality it will become more feasible and important to develop models based directly on these data, requiring semi-automated image processing pipelines to produce these geometries in both 2D and 3D. This will enable the models to be applied to more specific conditions including disease states, with many pathologies producing complex and heterogeneous subcellular remodeling. However, there are still numerous challenges in regards to model validation of Ca^2+^ spark spatio-temporal functional properties. Because of the difficulty in measuring Ca^2+^ function and underlying sub-cellular structure simultaneously, computational modeling can be useful to help fill in the gaps to relate structure to function, but for the same reasons, challenging to directly validate. It is worth highlighting that the models of single nanodomains do not reproduce well experimentally measured values for the full width half maximum (FWHM) of Ca^2+^, generally resulting in values of ∼1 μm which are below the 1.8–2.2 μm in experiment. Hoang-Trong et al. (2021) for example did simulate a realistic feature of 1.85 μm, but this required using a large RyR cluster combined with two smaller satellite clusters. However, due to spatio-temporal limits on the resolution of functional imaging experiments, sparks smaller than given sizes are not detected experimentally with any given accuracy. This generates an arbitrary experiment-dependent cut-off that affects the spark distribution and thus makes comparisons between simulation and experiment non-trivial.

A potential limitation of these models is the high spatial-resolution (and thus small voxel/element volumes) required to model RyR distribution: whereas this does not pose a problem during CICR, in which local Ca^2+^ concentration is relatively large, it does pose a problem at resting/quiescent Ca^2+^ levels, where the low Ca^2+^concentration of ∼0.1 μM in small volumes of 10^–21^–10^–18^L (Scriven et al., 2013) corresponds to the presence of countable numbers of Ca^2+^ ions (i.e., ion distribution is discrete and the notion of a well-defined concentration is debatable); similar considerations may also apply in the junctional SR during CICR when its Ca^2+^ load has been depleted (Hake et al., 2012; Cannell et al., 2013). Continuous approximations may therefore no longer be valid, and simulations of spontaneous Ca^2+^-sparks, in particular, may be non-trivial to implement and analyze. Hybrid schemes which implement spatial stochastic methods to capture the trajectories of individual particles, such as presented in skeletal muscle simulations implementing the Mcell framework (Kerr et al., 2008; Holash and MacIntosh, 2019), may offer solutions to this challenge.

These spatial nanodomain models have demonstrated the importance of specific RyR arrangement – and not just the total number – in determining Ca^2+^ spark dynamics. This presents a challenge for the translation to whole-cell modeling, as it is not feasible to simulate ∼20,000 spatially distributed dyads in a whole cell at resolutions of ∼10 nm, even if this high resolution is only adaptively applied in the local dyadic space. Therefore, in order to understand how a heterogeneous system of dyads with different RyR numbers and spatial arrangements coordinates in a whole-cell, coarse-graining methods will need to be developed which capture the features of this dyad heterogeneity at a reduced computational cost.

## Spatial Models of the Whole Cardiomyocyte

Spatial models of the cardiomyocyte describe the cell as a 2D area / 3D volume throughout which dyads are distributed and within which Ca^2+^ can diffuse in both the cytosolic and SR spaces. It is useful now to bring in the terminology of a Ca^2+^-release-unit (CRU). Whereas some studies use this to refer to the dyad or individual RyRs, cellular-scale modeling studies commonly refer to all of the intracellular and SR compartments associated with a single dyad as a CRU, i.e., it can be thought of as the entire volume of cell surrounding each dyad. Typically, this will contain the network SR (NSR), bulk cytoplasmic space (cyto), and the restricted volume of the dyad (treated as a common-pool). It is also common to include a distinct junctional SR compartment (JSR), as well as other optional sub-spaces, such as the sub-sarcolemma volume just below the surface or T-system membrane, included in order to preserve the higher local Ca^2+^ concentration close to a dyad in regions where the membrane fluxes are located. A major focus of this section is to discuss the various approaches to modeling sub-cellular structure in regards to spatial Ca^2+^ coupling.

### Fundamental Model Equations of Ca^2+^ Transport and Homeostasis

Calcium homeostasis in the compartments is described by:


(24) $$\frac{d{\left[Ca^{2+}\right]}_{cyto}}{dt}=\beta_{cyto}\left(\text{D}\nabla^{2}{\left[Ca^{2+}\right]}_{cyto}+\phi_{cyto}+\left(v_{ss}/v_{cyto}\right)J_{ss}\right)$$

(25) $$\frac{d{\left[Ca^{2+}\right]}_{SS}}{dt}=\beta_{SS}\left(\text{D}\nabla^{2}{\left[Ca^{2+}\right]}_{SS}+\phi_{SS}-J_{ss}+\left(v_{ds}/v_{ss}\right)J_{ds}\right)$$

(26) $$\frac{d{\left[Ca^{2+}\right]}_{nSR}}{dt}=\beta_{nSR}\left(\text{D}\nabla^{2}{\left[Ca^{2+}\right]}_{nSR}+\phi_{nSR}-\left(v_{jsr}/v_{nsr}\right)J_{jSR}\right)$$

(27) $$\frac{d{\left[Ca^{2+}\right]}_{ds}}{dt}=\text{D}\nabla^{2}{\left[Ca^{2+}\right]}_{ds}+\phi_{ds}-J_{ds}$$

(28) $$\frac{d{\left[Ca^{2+}\right]}_{JSR}}{dt}=\beta_{jSR}\left(\phi_{JSR}+J_{jSR}\right)$$

Transfer between compartments is given by:


(29) $$J_{ss}=\left({\left[Ca^{2+}\right]}_{SS}-{\left[Ca^{2+}\right]}_{cyto}\right)\tau_{ss}^{-1}$$

(30) $$J_{ds}=\left({\left[Ca^{2+}\right]}_{ds}-{\left[Ca^{2+}\right]}_{SS}\right)\tau_{ds}^{-1}$$

(31) $$J_{jSR}=\left({\left[Ca^{2+}\right]}_{nSR}-{\left[Ca^{2+}\right]}_{jSR}\right)\tau_{jSR}^{-1}$$

And the general form for the reaction terms are:


(32) $$\phi_{cyto}=J_{NaCa}+J_{pCa}+J_{Cab}-\left(J_{up}-J_{leak}\right)-J_{trpn}$$

(33) $$\phi_{nSR}=\left(J_{up}-J_{leak}\right)\left(v_{i}/v_{nsr}\right)$$

(34) $$\phi_{ss}=J_{NaCa\_SS}+J_{pCa\_SS}+J_{Cab\_SS}$$

(35) $$\phi_{ds}=J_{rel}+J_{CaL}$$

(36) $$\phi_{JSR}=-J_{rel}\left(v_{ds}/v_{jSR}\right)$$

Where *cyto*, *SS*, *nSR*, *ds*, and *JSR* refer to the Ca^2+^ concentrations in each of the (sometimes optional) compartments, β refers to the instantaneous buffering term, ϕ refers to a general reaction term in each compartment, *J*_*x*_ refers to transfer flux between compartments, ∇^2^ is the spatial Laplacian operator in 2D or 3D, describing coupling between CRUs, *D* is the diffusion constant, *v* refers to the volumes of the compartments, and τ to the time constants of diffusion. The concentration in the dyadic space can be described by a quasi-steady-state approximation, motivated by the rapid equilibration of Ca^2+^ in this small volume. By setting:


(37) $$\frac{d{\left[Ca^{2+}\right]}_{ds}}{dt}=0$$

An approximation for equation (27) can be obtained as in Hinch (2004):


(38) $${\left[Ca^{2+}\right]}_{ds}={\left[Ca^{2+}\right]}_{SS}+\frac{\tau_{ds}.\left(k_{rel}.{\left[Ca^{2+}\right]}_{jSR}+J_{CaL}\right)}{\left(1+\tau_{ds}.k_{rel}\right)}$$

Where *k*_rel_ is defined by *J*_rel_ = *k*_rel_(Ca_SR_-Ca_ds_) and therefore corresponds to:


(39) $$k_{rel}=n_{RyR\_O}.g_{RyR}.v_{ds}^{-1}$$

Where *g*_RyR_ is the conductance of a single RyR channel and *n*_RyR_O_ is the number of open RyRs in the dyad (corresponding to states O or O+O^*^, dependant on the RyR model implemented). This approximation enables less constraint on the time-step for the simulation, allowing faster simulations to be performed. A limitation of this approximation is that the introduction of this type of equation leads to a lack of ionic Ca^2+^ conservation in the models; similar issues arise with the implementation of the rapid buffering approximations [β terms in equations (24) – (28)]. Models that try to analyze homeostatic properties require that the computing algorithm conserves ions at all orders (Conesa et al., 2020).

### Ca^2+^-Voltage Coupling; Incorporation With an Action Potential Model

In general, it is only the Ca^2+^ concentrations and Ca^2+^-handling channels which are described spatially, with the membrane voltage (V_m_) and other ion-currents/concentrations assumed to be homogeneous throughout the cell. This is justified by the fast diffusion of V_m_ along the cell membrane, indicating that it is valid to assume all channels “see” the same global voltage, at least, in the time-frames considered by the models; moreover, the Debye length in cardiac cells is approximately 1 nm (Mori et al., 2008). Detailed electro-diffusion models, such as implemented in simulations of neurons (Pods et al., 2013), are therefore not generally used or required for cardiomyocytes. The spatial description of the Ca^2+^ handling system can simply replace the equivalent components of common-pool models and can therefore be integrated with either simplified descriptions of the AP (such as assuming it follows a simple, analytical form) or biophysically detailed models of the primary ion currents and global ionic concentrations.

The Ca^2+^ and voltage systems are coupled through the influence of V_m_ on the activity of the LTCCs and NCX (and any other voltage-dependent Ca^2+^ channel), and feedback of Ca^2+^ into the voltage is captured if a biophysically detailed model of the AP is included, wherein *I*_CaL_ and *I*_NaCa_ directly influence V_m_. Thus, the interaction between global voltage and local Ca^2+^ dynamics can be described in these models, enabling study of the mechanisms of, for example, Ca^2+^-induced AP duration (APD) alternans and afterdepolarizations (Eisner et al., 2009, 2017; Qu et al., 2014).

Whereas explicit diffusion of V_m_ along the membrane of a single-cell has generally not been included in the described models, this has been simulated in models presented by Crocini et al. (2014) and Scardigli et al. (2018). These studies simulated the impact of disruption of the T-system in disease on the ability of the AP to propagate along the T-system into the interior of the cell, indicating that AP propagation failure can directly contribute to a loss of intracellular Ca^2+^ synchronization.

### Model Discretization

**FIGURE 5:**
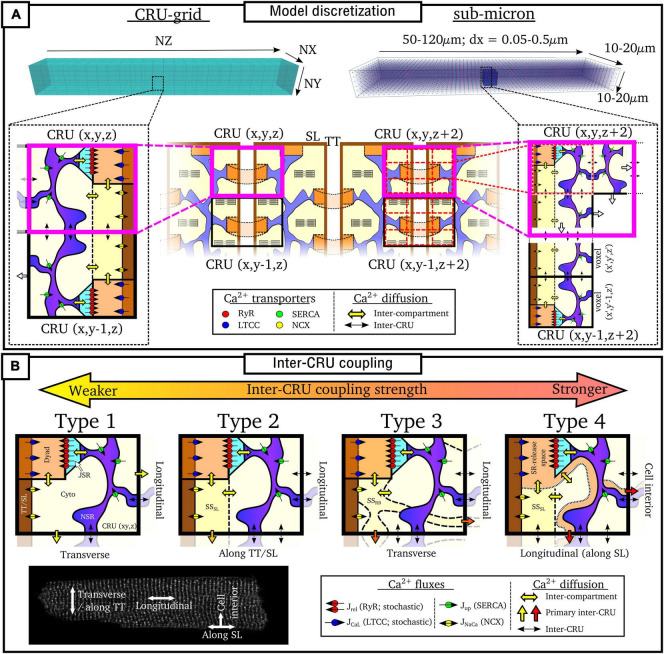
Discretization and CRU coupling of whole-cell models of spatial Ca^2+^ handling. **(A)** Illustration of the CRU-grid and sub-micron approaches to model discretisation. Top panels illustrate the whole-cell whereas bottom panels demonstrate the discretisation of the cell into either CRUs or sub-CRU voxels. **(B)** Illustration of different model structures for inter-CRU coupling. The primary pathway of CRU coupling in the intracellular space is shown by the colored-block arrows. Note that within the cell, coupling is symmetric in the ±-axes directions; a single direction only is shown for the purpose of clarity. Cell image for illustration provided by Dr. Izzy Jayasinghe, The University of Sheffield.

Numerical solutions to the spatio-temporal reaction-diffusion equations above require the cell to be described in a discretized space. Models can be broadly categorized as being one of two approaches (Figure 5A):

(1) “**CRU-grid**” or “**compartmentalized**” models, wherein the spatial resolution is ∼ 1 μm × 1 μm × 2 μm and each pixel/voxel corresponds to a single CRU;
(2) “**Sub-micron**” or “**free-diffusion**” models, wherein the volumes of the SR and cytoplasm, and SS if present, are discretized *within* each CRU.

Details of each approach are described below. In general, the CRU-grid approach is more computationally efficient and consequently suitable for statistical simulations and high-throughput, population-cohort models of spatial structure and heterogeneity. Its simplified structure also facilitates the dissection of the mechanisms by which various components contribute to macroscopic function. The advantages of the sub-micron approach are that one can more precisely control aspects such as heterogeneous inter-dyad spacing and co-localization of different channels, they have a more accurate recapitulation of Ca^2+^ diffusion and cellular geometry, and are better suited to direct incorporation of experimental imaging data.

#### Ca^2+^-Release-Unit-Grid Models

In these models, each voxel contains all of the compartments contained within a CRU. Spatial coupling is described using the isotropic finite-difference method (FDM), or approximations thereof. Each CRU is coupled to its four or six nearest-neighbors (for 2D or 3D, respectively) along the principal axes:


(40) D∇2[Ca2+]c=cyto,nSR,(SS)≈βDdeix2∑i=1i=3([Ca2+]cei+1+ei-1[Ca2+]c-2[Ca2+]cei)

Where *e*_i_ refers to the three dimensions (x,y,z), the subscript *c* refers to any compartment which is spatially coupled (i.e., the bulk cyto and NSR spaces and any coupled sub-space), *D* is the diffusion coefficient and *dx* is the spatial step (i.e., the resolution). The diffusion term (D/dx^2^) is often approximated with a time-constant of diffusion between spatially coupled compartments (τ_*c,ei*_):


(41) D∇2[Ca2+]c≈JCa_diff_c=∑i=1i=3([Ca2+]cei+1+ei-1[Ca2+]c-2[Ca2+]ceiτc,ei)

Note that whereas these approximations are derived from the isotropic FDM, the models are often discretized at a larger resolution in the longitudinal direction than the transverse, reflecting the larger spacing of dyads along the cell compared to along a TT. Thus, *dx*^2^ in equation (40) or τ in equation (41) can be larger in the longitudinal (z) direction compared to the transverse. This therefore introduces an anisotropy (preferential propagation along the transverse direction) despite the model equations being derived from isotropic approximations.

#### Sub-Micron Models

In these models, each CRU is further discretized into voxels or elements. Not every voxel necessarily contains all compartments. For example, nanodomains/jSR will only be present in a small subset of voxels, and in some models, the T-system or even nSR may also only be present in a subset of voxels. Spatial coupling is solved using FDM [equation (40)] on regular structured grids or using the more complex finite element method (FEM) on structured or unstructured meshes. They can be discretized at different choices of resolution, generally between 0.05 and 0.2 μm (Nivala et al., 2012a,^2015^; Colman et al., 2017b; Marchena and Echebarria, 2018, 2020; Hoang-Trong et al., 2021), although there are other intermediary approaches, such as Sutanto et al. (2018) which uses the CRU-grid approach in the transverse direction but is discretized at half-CRU distance in the longitudinal direction.

### Model Compartment Structure: Inter-Ca^2+^-Release-Unit Coupling

In addition to model discretization, there are important differences in underlying model structure, notably regarding the mechanisms of inter-CRU coupling. Different structures of inter-CRU coupling lead to variable strengths of spatial Ca^2+^ coupling, in part due to the variable peak Ca^2+^ concentrations in the compartments selected for coupling: compartments which have a smaller volume and are more directly coupled to the dyad exhibit larger CaTs and thus stronger inter-CRU coupling compared to larger compartments which are less directly coupled to the dyad. In this context, coupling strength refers only to the intracellular space, not the SR, the spatial coupling of which is independently controlled. Broadly, there can be considered four different structures which the models follow (Figure 5B and Table 1):

**TABLE 1:** Summary of properties of whole-cell models of spatio-temporal Ca^2+^ handling.

Publication	CRU-coupling	Discretisation	T-system	Celltype
Alvarez-Lacalle et al., 2015	Type 2	CRU-grid	Full	Ventricle
Colman, 2019	Type 3	CRU-grid	Full	Generic
Colman et al., 2016	Type 3	CRU-grid	Variable	Atria
Colman et al., 2017a	Type 3	CRU-grid	Full	Ventricle
Colman et al., 2017b	Type 3	Sub-micron	Realistic	Ventricle
Conesa et al., 2020	Type 2	CRU-grid	Full	Ventricle
Gaur and Rudy, 2011	Type 4	CRU-grid	Full	Ventricle
Hoang-Trong et al., 2021	Type 1	Sub-micron	Detailed	Ventricle
Greene and Shiferaw, 2021	Type 2	CRU-grid	Variable	Atria
Marchena and Echebarria, 2018, 2020	Type 1	Sub-micron	Variable	Atria
Nivala et al., 2015	Type 1	Sub-micron	Variable	Ventricle
Nivala et al., 2012a,b	Type 1	Sub-micron	Full	Ventricle
Restrepo et al., 2008	Type 2	CRU-grid	Full	Ventricle
Sato et al., 2016	Type 2	CRU-grid	Full	Ventricle
Shiferaw et al., 2017, 2018, 2020	Type 2	CRU-grid	Variable	Atria
Singh et al., 2017	Type 2	CRU-grid	Variable	Ventricle
Song et al., 2015, 2016, 2017	Type 2	CRU-grid	Full	Ventricle
Song et al., 2018	Type 2	CRU-grid	Variable	Generic/Ventricle
Song et al., 2019	Type 2	CRU-grid	Variable	Ventricle
Sutanto et al., 2018	Type 4	Hybrid	Variable	Atria
Vagos et al., 2020, 2021	Type 4	CRU-grid	None	Atria
Voigt et al., 2014	Type 4	CRU-grid	None	Atria
Williams et al., 2011	Type 1	CRU-grid	Full	Generic/Ventricle

#### Type 1

The simplest model structure comprises of the four compartments of the bulk cytoplasm and dyadic space and network and junctional SR. The bulk cytoplasm and network SR spaces are the only ones which are spatially coupled throughout the cell. These models contain the weakest coupling due to the coupling of bulk cytoplasm only, which contains Ca^2+^ concentrations of the same order of magnitude as the whole-cell average. Models using this include Nivala et al. (2012a,b, 2015).

#### Type 2

Many models also include a sub-sarcolemmal subspace from/into which the other membrane fluxes (NCX and the plasmalemmal Ca^2+^ pump) act. This subspace has a smaller volume and higher Ca^2+^ concentrations at peak than the bulk cytoplasm. This sub-space is generally coupled between CRUs in the transverse direction only, i.e., along the TTs. Longitudinal coupling of this sub-space may be present where ATs are modeled, but does not by default occur for every CRU throughout the cell. Coupling strength is higher than the simplest type 1 models due to the larger CaT in this sub-space. Models of this type include Song et al. (2015, 2016, 2018).

#### Type 3

Other models implement a sub-space which couples CRUs in both transverse and longitudinal directions independently of the presence or absence of T-system/SL. Introduced in Colman et al. (2017a,b) and Colman (2019), this sub-space contains fewer buffers and represents potential pathways between dyads around the intracellular buffers. Given the reduced buffering (higher CaT peaks) and coupling in all directions, these models have stronger inter-CRU coupling than those which contain a sub-sarcolemmal subspace only.

#### Type 4

Finally, other models have more direct inter-CRU coupling between dyads, or SR-release spaces (Gaur and Rudy, 2011; Voigt et al., 2014; Sutanto et al., 2018; Vagos et al., 2020). Due to this direct spatial coupling of the compartment into which release occurs, these models have the strongest spatial coupling.

This classification is simplified, but captures the major features of the various approaches. For example, the Heijman-lab models (type 4) were originally designed to represent atrial cells with no T-system: interior compartments did not contain the SL fluxes or associated sub-space. However, models of type 1–3 can be generalized to match this structure by removing these same SL fluxes and associated sub-space (where present) from interior CRUs, e.g., as in Colman et al. (2016), Shiferaw et al. (2017), Song et al. (2018), and Marchena and Echebarria (2020). Similarly, the Heijman-lab models can also be generalized to incorporate a T-system by the inclusion of the SL fluxes and sub-space in interior CRUs, e.g., as was performed in Sutanto et al. (2018). Thus, the above types represent four fundamentally different approaches to inter-CRU coupling, and can be generalized to any cell structure, containing, for example, full, no, or variable T-system density (see next section “Modeling variability in sub-cellular structure and function”).

### Implications of Model Structures

One major feature of the differences between these models is the relationship between RyR sensitivity, inter-CRU coupling strength and the size of the CaT in normal pacing, which has significant implications for the robustness of inter-CRU Ca^2+^ propagation. Broadly, models with weaker inter-CRU Ca^2+^ coupling (type 1–2 above) tend to contain either a physiologically sized CaT and operate at the threshold of Ca^2+^ propagation, or contain a substantially larger CaT (>2–8 μM) with more robust Ca^2+^ propagation (Nivala et al., 2015; Song et al., 2015; Marchena and Echebarria, 2020). Alternatively, widespread initiation of CICR (reflecting an extensive T-system bringing LTCC close to RyR throughout the cardiomyocyte) contributes to robust, synchronized Ca^2+^ release in some of these models. Models with stronger inter-CRU coupling (type 3–4 above) tend to contain more robust Ca^2+^ propagation while maintaining physiologically sized CaTs (i.e., below 1 μM), as in Voigt et al. (2014), Colman et al. (2017a), Colman (2019), and Vagos et al. (2020). Such robust propagation is particularly relevant when simulating cells without an extensive T-system that rely more heavily on fire-diffuse-fire mechanisms for Ca^2+^-wave propagation, such as in the atria. The implications of these differences are far-reaching: Ca^2+^ propagation is relevant for graded release of CICR, the dynamics of CaT alternans, success or failure of triggered Ca^2+^ wave propagation into regions without T-system, and the dynamics of spontaneous Ca^2+^ sparks and waves.

These features and limitations, generally discussed openly in the original papers, do not detract from the ambitions of the various studies nor question their analyses, as model choices are motivated by the focus of the specific study. It is important to note that all approaches are capable of reproducing all of the phenomena described above, including properties such as the statistics of SCRE and dynamics of alternans, although various model parameters are likely to be substantially different in order to reproduce these same macroscopic features. Nevertheless, it is still important to carefully consider the motivations of the study for model selection. As an oversimplified example, if the ambition is to study Ca^2+^-voltage interactions during SCRE or alternans, it may be best to prioritize CaT magnitude (as this will determine the degree of Ca^2+^-induced inactivation of *I*_CaL_ and the magnitude of *I*_*NCX*_ which feedback into the voltage) and use a more “functional” description of inter-CRU coupling (types 3–4) in order to maintain robust Ca^2+^ propagation. However, if the focus of the study is on the mechanisms and implications of inter-CRU coupling then the simpler and (potentially) more physiologically justified inter-CRU coupling structures (types 1–2) may be prioritized. These considerations may be particularly relevant for the development of atrial cell models and those with variable T-system density, and will be discussed in this context in more detail in the next section.

These disparities and compromises indicate that there are fundamental properties of the CaT and inter-CRU coupling which we do not fully understand, and these gaps in our understanding and model differences reflect the large degree of uncertainty in the experimental data on which the models are based. There is an important possibility that different internal structures need different behavior at the dyadic level since weaker coupling generally requires stronger transients to reproduce wave-like propagation. This might lead to important model-dependent conclusions on new research issues. As models become more sophisticated, getting closer to genuine cell- and species- specificity (indeed as more data become available), and are applied in more complex and clinically oriented studies, it will become imperative to solve these issues and develop models which include fully physiologically justified descriptions of RyRs and spatial Ca^2+^ coupling. Further to this, it is these authors’ opinion that future studies would do well to implement multiple, disparate models in order to navigate the limitation of model-dependent conclusions.

## Modeling Variability in Sub-Cellular Structure and Function

Cardiomyocytes demonstrate a large degree of inter-cellular, inter-subject and inter-species heterogeneity in properties such as ion-channel expression and sub-cellular structure, and recent studies have highlighted the importance of including such variabilities in, for example, predictive models of pharmacology (Muszkiewicz et al., 2016; Passini et al., 2017). This section will describe how heterogeneity in sub-cellular structure can be captured using models of spatial Ca^2+^ cycling.

### T-System Variability: Models of Atrial and Remodeled Ventricular Myocytes

**FIGURE 6:**
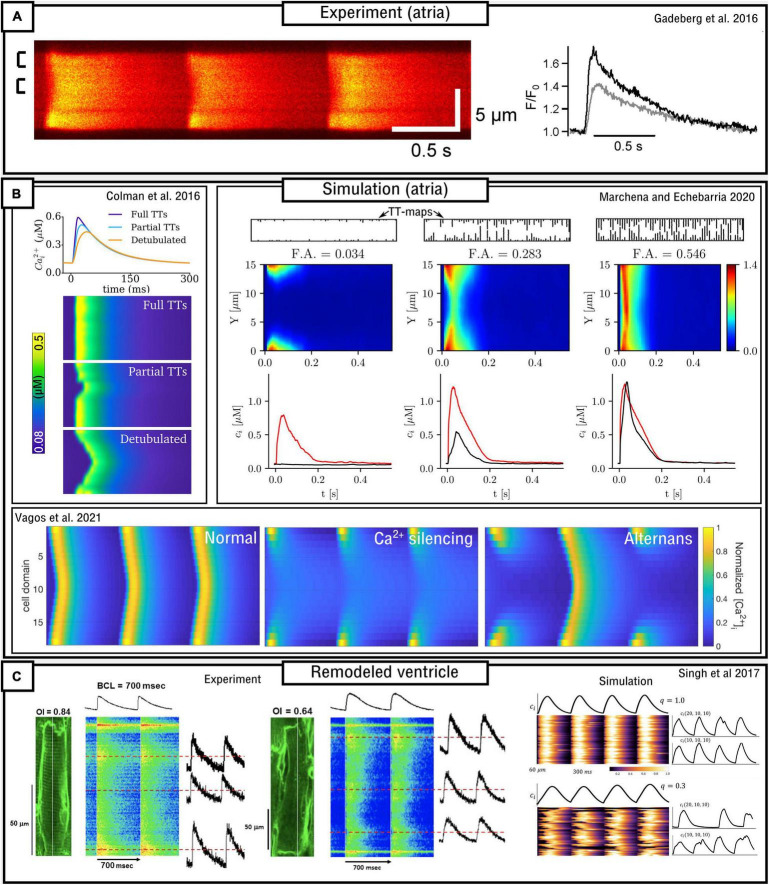
Models of variable T-system density in atrial and remodeled ventricular myocytes. **(A)** Experimental data demonstrating “u” waves in pig atrial myocytes, and the corresponding local CaTs at the surface (black) and interior (gray) of the cell. Data is from Gadeberg et al. (2016). **(B)** Dynamics in different computational models of atrial cells, illustrating recapitulation of “u” waves, dependence on T-system density, and Ca^2+^ silencing and CaT alternans. Data from Colman et al. (2016); Marchena and Echebarria (2020), and Vagos et al. (2021). **(C)** Experimental and simulation data from remodeled ventricular myocytes (Singh et al., 2017), demonstrating a loss of T-system density and associated loss of spatial synchrony of the CaT, in relation to the Organizational index (OI; experiment) and the proportion of CRUs associated with LTCCs (q; simulation).

The structure and density of the T-system is one of the most important factors which determines sub-cellular dynamics. In healthy ventricular myocytes the T-system is generally robust and dense throughout the volume of the cell. However, in atrial myocytes and diseased ventricular myocytes the T-system can be substantially sparser and more variable (Lyon et al., 2009; Richards et al., 2011; Gadeberg et al., 2016; Singh et al., 2017). This reduction in T-system density is generally correlated with alterations to the CaT (primarily, a prolonged time-to-peak, reduced spatial synchronization, and often a small, sometimes large, reduction in magnitude) and possibly linked to an increased vulnerability to pro-arrhythmic dynamics (Trafford et al., 2013; Gadeberg et al., 2016; Shiferaw et al., 2017, 2018). In these conditions, triggered Ca^2+^ sparks will occur only in regions of the cell where LTCCs are closely coupled to RyRs [although even these regions may not exhibit triggered sparks if the AP fails to reach them (Crocini et al., 2014)]. Ca^2+^ may then propagate into regions without the T-system, i.e., where orphaned RyRs are found without coupling to the LTCCs, through spark-induced-spark triggered Ca^2+^ waves, resulting in “u” or “w” shaped linescan images (Figure 6). However, Ca^2+^ may also fail to propagate as a triggered wave, leading to regions of the cell which do not undergo substantial Ca^2+^-release which consequently underlies a substantially smaller whole-cell CaT. The conditions which either enable or inhibit triggered Ca^2+^-wave propagation, which may be species-, cell-, disease-, and environment-dependent, are unclear from experimental studies alone, and these analyses form the focus of many computational studies which implement variable T-system density and structure.

Implementation of variable T-system structure is relatively straight-forward in these spatial models of Ca^2+^ handling, for both the CRU-grid and sub-micron approaches. The inclusion or omission of a TT or AT from a CRU or voxel/element can be trivially implemented by either the inclusion or omission of the membrane Ca^2+^ fluxes (LTCCs, NCX, Ca^2+^ pump) and any associated sub-sarcolemma sub-space. Thus, one needs only create a map which describes which CRUs or voxels/elements contain a TT or AT. Creation of this map could be through random selection, a T-system generating algorithm, or directly based on experimental imaging data.

#### Modeling Atrial Cardiomyocytes

Atrial cells exhibit variable T-system density in both control and disease conditions (Richards et al., 2011; Park et al., 2020), and thus computational models of atrial cells tend to either not include the T-system or explicitly model its variability. Koivumäki et al. (2011) developed a simplified, 1D model of the atrial myocyte which captured the propagation of Ca^2+^ waves from the surface to the interior. The deterministic model contained four sub-cellular compartments as a coarse-grained discretisation of the transversal direction of the cell. Colman et al. (2016) implemented an atrial cell model with variable T-system density in a “type 3,” 3D CRU-grid model. Variable T-system structure was crudely implemented by removing patches of the T-system of variable and controllable size within the cell, based on randomly generated seeds. This model demonstrated “u” and “w” linescans during normal pacing (Figure 6), a delay in the time-to-peak, and a small reduction in the magnitude of the CaT as T-system density decreased. CaT alternans and SCRE were found to increase as T-system density decreased and the specific dynamics were highly controlled by the structure: alternans involved alternating between successful and failed Ca^2+^ propagation into the non-T-system regions, and spontaneous Ca^2+^ waves preferentially emerged from regions without a TT or AT.

The atrial models developed in the Heijman-lab (Voigt et al., 2014; Vagos et al., 2020) contained no T-system by default. These models, of type 4 structure, demonstrate generally robust Ca^2+^ propagation into the cell interior, the mechanisms and sensitivity of which were investigated in detail in Vagos et al. (2020) and further expanded on in Vagos et al. (2021). A mechanism of CaT alternans similar to that described in Colman et al. (2016) was observed (Figure 6). Sutanto et al. (2018) expanded the approach by incorporating RyR/LTCC expression/distribution based on experimental imaging data (see later sub-section “Pipelines for image-based modeling”), as well as the inclusion of variable T-system in the interior of the cell, demonstrating the important role of both TTs and ATs in facilitating Ca^2+^ propagation.

In a series of studies which integrated computational modeling with experimental functional measurements of atrial cell dynamics Shiferaw et al. (2017, 2018, 2020) comprehensively investigated the mechanisms and sensitivity of triggered Ca^2+^ wave propagation using a model of heterogeneous T-system density, in combination with other factors such as β-adrenergic stimulation. In brief, these studies demonstrated rate-dependent triggered Ca^2+^ waves emerging in SR-Ca^2+^ overload conditions. It is argued that the emergence and dynamics of triggered Ca^2+^ waves exhibit a highly non-linear dependence on SR-Ca^2+^ load and that this feature of atrial cells contributes to dynamic instabilities which may be pro-arrhythmic.

Marchena and Echebarria (2018, 2020) also developed models of the atrial cell using a type 1 sub-micron approach. A comprehensive analysis of the relationship between the fractional area occupied by the T-system and the magnitude and morphology of the whole-cell CaT was provided (Figure 6). These studies indicated that SR-Ca^2+^ load was unaffected by T-system density and that reduced CaTs were a consequence of failure of Ca^2+^-wave to fully propagate from the cell periphery into the interior; however, Ca^2+^-release gain was increased in detubulated cells as a consequence of the activation of at least some orphaned RyRs. No significant differences in spontaneous Ca^2+^ spark dynamics were found, indicating that experimental observations of heterogeneous spark dynamics (Hüser et al., 1996; Kirk et al., 2003; Brette et al., 2005) could be explained by different kinetics or regulation of RyRs in membrane and non-membrane regions, not captured in the models. This is consistent with Brandenburg et al. (2016) and Sutanto et al. (2018) which show, in experiment and modeling, that RyR hyperphosphorylation contributed to these regional differences.

#### Modeling Ventricular Cardiomyocytes With Disease-Related T-System Remodeling

In a combined experimental-computational study, Wagner et al. (2012) characterized the remodeling of sub-cellular structure (T-system properties and other related proteins such as junctophilin) post myocardial-infarction (MI). They observed a progressive, time-dependent post-MI increase in the cross-sectional area of individual T-tubules, a decreased expression of junctophilin, an orphaning of RyR clusters, and uncoupling of CICR. Simulations were performed using the previously presented model of Williams et al. (2011), which implemented the simplest type 1 structure at CRU-grid resolution. Remodeling was incorporated by an increased spacing between TTs and RyRs in a subset of the model’s compartments. Simulation results demonstrated that RyR orphaning contributed to post-MI associated AP prolongation, especially when combined with remodeling of NCX and SERCA, as well as reduced SR-Ca^2+^ load and increased Ca^2+^-leak, potentially contributing toward arrhythmogenic afterdepolarizations. However, the changes to TT cross-sectional area were not captured in this model and require more detailed models, discussed in the later sub-section: “Toward realistic sub-cellular structure.”

Nivala et al. (2015) implemented a loss of the T-system representing remodeled ventricular myocytes using a sub-micron, type 1 model. Voxels containing LTCCs (i.e., dyads) were randomly selected for removal of the membrane components. This study demonstrated that disruption of the T-system led to perturbed spatial Ca^2+^ handling (delay in the time-to-peak and slightly reduced magnitude of the CaT) which was more pronounced when combined with remodeling of whole-cell parameters associated with heart failure.

Song et al. (2018) expanded on the work of Nivala et al. (2015) using a type 2 CRU-grid model. In this study, an algorithm was developed to generate more realistic variable T-system structures. Results were largely concurrent with the previous studies, with the major differences that: (1) a more substantial reduction in CaT amplitude was observed in detubulated cells; (2) a biphasic relationship between T-system density and arrhythmogenic dynamics was observed, wherein intermediate densities demonstrate the most instabilities.

Singh et al. (2017) combined experimental measurements and computational modeling to explore the relationship between T-system density and the features of the CaT in a rat ventricular model of the progression of heart failure (Figure 6C). Experimental measurements, in agreement with their previous study (Shah et al., 2014), demonstrate that the CaT exhibits a slower upstroke and reduced magnitude associated with substantial loss of the T-system, but Ca^2+^ propagation is not silenced. The computational (type 2) model was in strong agreement with these observations, revealing a non-linear relationship on both distribution of release units and separation between LTCCs and RyRs.

#### Model Parameterization

Differences in the dynamics and sensitivity of triggered wave propagation observed in the models can be largely related to the underlying model structure (type 1–4 as described in the previous section). The development of models of atrial myocytes presents a major challenge in this context, i.e., in obtaining robust triggered Ca^2+^ wave propagation with physiologically sized CaTs but without an over-propensity for spontaneous activity. In the Colman-lab and Heijman-lab models, stronger inter-CRU coupling (type 3–4) was implemented for this purpose, as it facilitated triggered Ca^2+^ wave propagation with physiologically sized CaTs. Without this strong inter-CRU coupling, it was not possible to reproduce stable Ca^2+^ homeostasis during regular pacing while maintaining robust Ca^2+^ wave propagation and physiological CaTs without also observing highly arrhythmogenic spontaneous activity – either the spontaneous spark rate or Ca^2+^-wave rate was too high. Type 3–4 models somewhat solve this through the inclusion of these stronger coupling mechanisms, but also tend to exhibit low spontaneous spark rates and a higher probability for a spark to develop into a full wave. This indicates the possible physiological relevance of stronger coupling (e.g., pathways between buffers and/or along the SR membrane), but this has not been supported with imaging experiments.

It is generally assumed triggered waves should propagate in normal conditions in healthy atrial myocytes, but the validity of this assumption and whether it also translates to remodeled ventricular myocytes is not clear. It is worth noting that different experimental observations cover the range of behavior exhibited by these models, with some experiments observing generally robust triggered Ca^2+^-wave propagation with “u” or “w” waves and only a small reduction in the magnitude of the CaT in cells with low T-system density (Louch et al., 2010; Trafford et al., 2013; Crocini et al., 2014; Gadeberg et al., 2016; Setterberg et al., 2021), whereas others demonstrate more variability and sensitivity of triggered Ca^2+^ waves in many different pacing conditions, associated with substantially smaller CaTs in detubulated cells in normal pacing conditions (Brette et al., 2005; Shiferaw et al., 2017).

### Toward Realistic Sub-Cellular Structure

#### Modeling Cellular Contraction, Mitochondria and Energetics

Two components of the structure-function relationships governing cardiac cellular electrophysiology which have not yet been discussed in detail are the myofilaments (and related contractile apparatus) and the mitochondria (and associated localized buffering and energetics). Okada et al. (2005) developed a FEM-based model of cellular contraction associated with intracellular Ca^2+^ waves, enabling investigation of the impact of cell shortening on Ca^2+^-wave velocity. The model also revealed the potential for spiral Ca^2+^ waves which could maintain arrhythmicity. Hatano et al. (2011, 2012) expanded this model to include realistic local mitochondrial Ca^2+^ buffering and ATP production, T-tubules, SR structure, and myofilaments. These models revealed slow changes in the average mitochondrial Ca^2+^ during the cardiac cycle and that asynchronous contraction caused by a large detubulated region can lead to impairment of myocyte contractile efficiency. Recently, Xie et al. (2018), Song et al. (2019), and Pandey et al. (2021) developed CRU-grid spatial models which also explicitly accounted for local buffering and dynamics of the mitochondria. These mitochondria were assigned to alternating CRUs in the transverse direction (but every CRU in the longitudinal direction) and the models were applied to study the role mitochondria may play in proarrhythmogenic dynamics including afterdepolarizations.

#### Heterogeneous Channel Distribution

Beyond the T-system, the distribution and local density of different Ca^2+^-handling transporters is also important for governing Ca^2+^ homeostasis through effects on local Ca^2+^-flux balance. Sub-micron models are ideal for controlling the fine details of channel distribution and co-localization between different channels, but CRU-grid models remain suitable for investigating heterogeneous channel/transporter expression in different regions of the myocyte.

The number of RyRs and LTCCs per dyad, as well as dyad volume, are commonly heterogeneous in the default implementations of many models (assigned by scaling the expression or volume by numbers randomly sampled from a normalized Gaussian or other distribution). These heterogeneous properties have been shown to be important for capturing the features of graded intracellular Ca^2+^-release (Greenstein and Winslow, 2002; Shiferaw et al., 2003; Restrepo et al., 2008). In principle, heterogeneous expression of any transporter or component (e.g., SERCA, NCX, buffer concentration) could be implemented in the same manner by sampling scale-factors from a defined statistical distribution. However, determining heterogeneous structure based on experimental data and with constraints on the spatial variation/correlation provide more powerful and physiologically relevant approaches.

#### Pipelines for Image-Based Modeling

**FIGURE 7:**
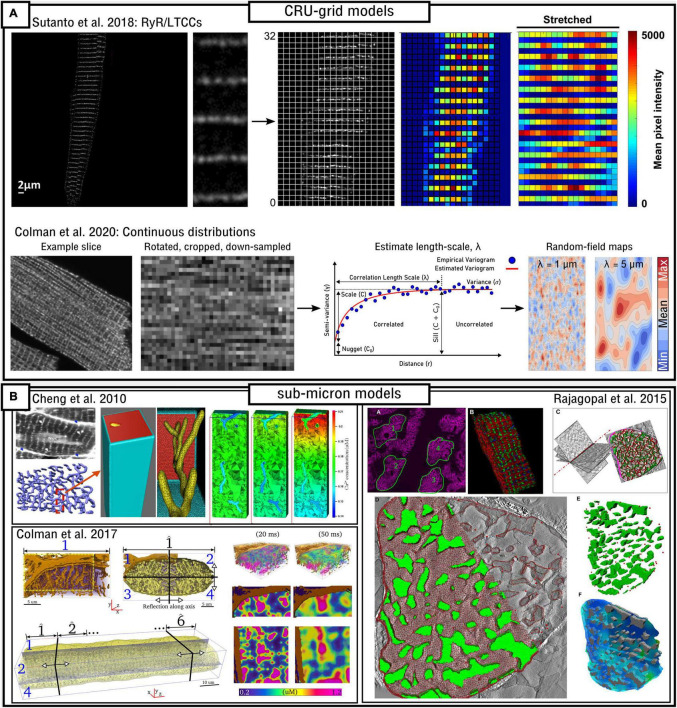
Image-based modeling approaches. **(A)** Approaches for image-based modeling in idealized, CRU-grid cell models. The top panel illustrates the processing of LTCC/RyR image data so that it can be aligned and registered on an idealized grid computational model, as presented in Sutanto et al. (2018). The lower panel illustrates the method of calculating the correlation length-scale (λ) in order to generate Gaussian-random field maps which match these parameters in order to describe sub-cellular heterogeneity, as presented in Colman et al. (2020). **(B)** Approaches for directly incorporating imaging data into realistic, sub-micron cell models. The top-left panel demonstrates processing of ultra-structural data describing T-system structure into a mesh for simulations, and illustrates the large Ca^2+^ gradients that can occur under different flux-distributions, as presented in Cheng et al. (2010). Lower panel illustrates processing of data describing the T-system and SR in a portion of a cell, approaches to tesselate the cell portion into a whole-cell, and the substantial Ca^2+^-gradients observed in this model also, as presented in Colman et al. (2017b). Right panel illustrates the semi-automated pipeline for segmenting multiple structures, including the myofibrils and mitochondria, into a 3D structurally-detailed representation of a sarcomere, as presented in Rajagopal et al. (2015).

In Sutanto et al. (2018) a pipeline was developed which enabled the expression of RyRs and LTCCs (and in principle any desired Ca^2+^-handling component) observed in experimental imaging studies to be processed to align with the modified CRU-grid model (Figure 7A). Thus, RyR or LTCC expression in each dyad was determined by the intensity of immunofluorescence image labeling for each of these channels. The method involved processing the real cellular data so that it could be registered on the idealized cellular geometry of the computational model.

In an alternative approach, a method was presented in Colman et al. (2020) which involved the development of image-analysis techniques to extract parameters describing the spatial correlation and distribution of the channels (Figure 7A). This involved calculating the length-scale which describes the distance over which expression is correlated. This parameter can be used to generate Gaussian random fields which produce expression maps with the same spatial correlations, but not limited to the specifics of the imaging data.

The above approaches enable efficient and high-throughput simulations of variable cellular structure to be performed, which are ideally suited to statistical analysis and the extraction of fundamental mechanisms of homeostasis. However, sub-micron models, which are substantially more computationally intensive, provide more possibilities for the direct inclusion of experimental imaging data. In a series of studies (Cheng et al., 2010; Kekenes-Huskey et al., 2012; Hake et al., 2014) an ultra-structure model of a realistic single TT and its surrounding half-sarcomeres was developed (Figure 7B), extending their previous work which implemented an idealistic TT (Lu et al., 2009). The local cellular geometry was reconstructed from light-and electron-microscopy images of a rat ventricular myocyte and the studies investigated the impact of the distribution of Ca^2+^-flux channels and buffers. They observed substantial and rapid spatial gradients in local Ca^2+^ concentration in the sub-sarcolemmal sub-space (Figure 7B) and their analyses demonstrate the importance of accounting for T-system ultra-structure and Ca^2+^ flux distribution. These detailed models present the possibility for features of T-tubule ultrastructural remodeling to be included, such as observed in Wagner et al. (2012) and Crocini et al. (2014), and could also potentially be extended to account for other local ionic concentrations and the counter-ion fluxes relevant for SR-Ca^2+^ release, such as presented in Berti et al. (2017).

In Colman et al. (2017b) a sub-micron, whole-cell model was developed which directly incorporated imaging data describing the structure of the T-system, SR and distribution of the dyads (Figure 7B). A semi-automatic pipeline was developed in which the images were processed and down-sampled to create simulation-ready geometries. Whereas only a portion of the cellular geometry was reconstructed, the model was tessellated to construct a whole-cell, exploiting the periodic structure of cardiac myocytes. This model also observed substantial intracellular Ca^2+^ gradients which emerged only in the realistic geometries, in agreement with Cheng et al. (2010). The imaging data was based on electron-microscopy (Pinali et al., 2013) and thus channel distribution was not captured in the data. However, future studies could use advances in correlative light-electron microscopy to integrate T-system and SR structure with immunofluorescence-based protein levels associated with each membrane, enabling the possibility to directly impose local relative channel expression on these structures.

This model also indicated the importance of realistic (and variable) dyad distribution on the specific dynamics of both CaT alternans and SCRE, providing spatial constraints on the randomness of both. These results are further supported by the recent sub-micron, type 1 model of Hoang-Trong et al. (2021) which included experimentally influenced spatial distributions of multiple Ca^2+^-flux channels, explicit modeling of transverse- and axial-tubules, and Ca^2+^-CaM interactions. This study highlighted the importance of CRU distribution and the presence of rogue (non-junctional or orphaned) RyRs on Ca^2+^-spark propagation and wave dynamics. The model was able to reproduce regenerative Ca^2+^ waves at high Ca^2+^-overload conditions, emerging from the same location on subsequent instances, and the implementation employed several techniques to reduce computational load and memory requirements, enabling efficient implementation of GPU solvers.

Rajagopal et al. (2015, 2018), Ghosh et al. (2018), and Hussain et al. (2018) present a rather different approach and focus to the other whole-cell computational models so-far described, with a higher level of structural detail accounted for Figure 7B. The 3D computational model is generated from images of the myofibrils, mitochondria and RyR clusters; Data from different sources and of different resolutions (e.g., 3D electron microscopy and high-contrast confocal) were fused through spatial statistics techniques (Illian et al., 2008; Theakston et al., 2010). These models focus on many more details of the structure of myocytes and their impact on local Ca^2+^ buffering and regulation, including that of the contractile apparatus and mitochondria, and simulations focus more on the upstroke of the CaT than on long-term homeostasis. The methods for processing high resolution imaging data to generate meshes for simulation are the most advanced in the field, and the potential of these models to understand the super-resolution features of channel distribution, co-localization and Ca^2+^ regulation is currently unparalleled. It would be a significant achievement to develop computational models which account for this level of structural detail that are also sufficiently efficient to simulate long-term dynamics, homeostasis, and Ca^2+^-voltage coupling in a whole-cell.

## Simplified, Minimal, and Tissue Models

**FIGURE 8:**
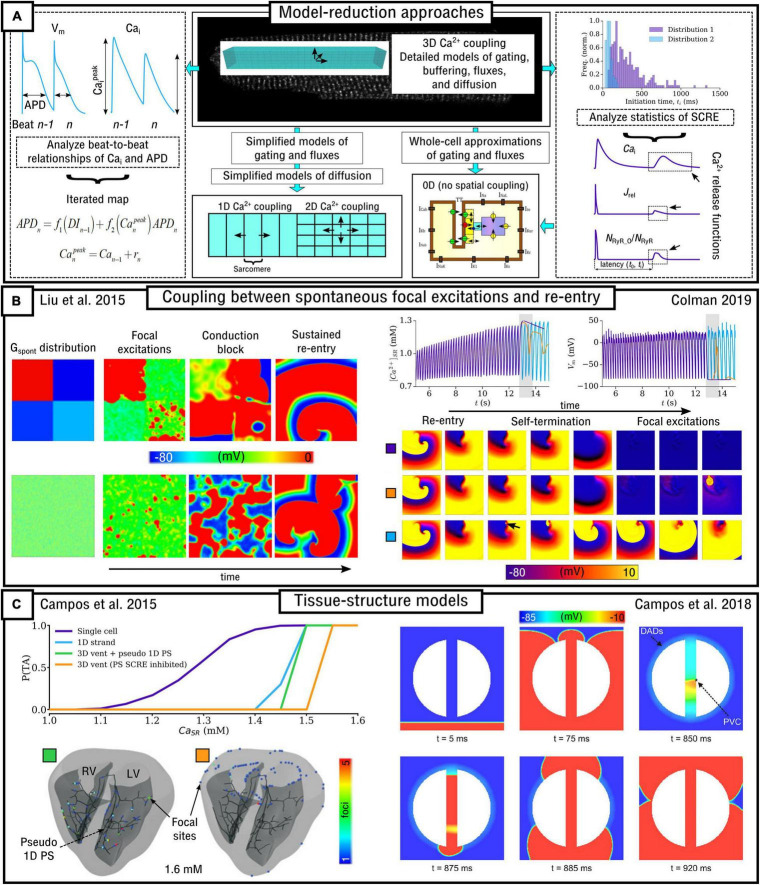
Multi-scale modeling approaches. **(A)** Illustration of different approaches for reducing the computational intensity of models of sub-cellular Ca^2+^ handling. The center, upper panel illustrates a fully detailed 3D cell model, with biophysically detailed descriptions of gating, fluxes and diffusion. Below this panel are illustrated mathematical approaches for model reduction, either resulting in simplified spatial models (left) or non-spatial models (right). The left-most panel indicates how analysis of AP and CaT dynamics on a beat-to-beat basis can lead to the development of a non-spatial, iterated map model. The right-most panel illustrates how analysis of SCRE statistics enables the parameterization of analytical Ca^2+^ release functions which can be imposed in a traditional, non-spatial cell model. **(B)** Illustration of bi-directional coupling between focal excitations and re-entry. Left: spontaneous excitations interact with each-other to cause conduction block and re-entry, modified from Liu et al. (2015). Right: illustration of the emergence of focal excitations following self-termination after a period of re-entry, from Colman (2019). **(C)** Structurally detailed tissue models imeplementing SCRE. Left: probability of SCRE as a function of SR-Ca^2+^ in single-cell and 1D, 2D, and 3D with or without the pseudo-1D Purkinje System (PS), with the locations of focal exictations shown in the lower panel, modified from Campos et al. (2015); right: demonstration of a focal excitation emerging within the isthmus of an infarct borderzone, modified from Campos et al. (2018).

The complexity of the spatio-temporal models described above hinders ease of analysis and extraction of fundamental mechanisms, and also precludes the efficient cellular simulations required to model hundreds, thousands or millions of cells in cardiac tissue, due to the high computational load of these detailed models. Thus, approaches are required to simplify these complex descriptions into easily analyzable systems and/or efficient computational models while preserving the underlying stochastic dynamics and the emergent phenomena therein. Almost 20-years ago, Shiferaw et al. (2003) presented a number of simplifications to describe spatial Ca^2+^ handling which underlie many of the developments since. Various different approaches have been used including those which explicitly model the cell as a spatial structure but with simplified components of Ca^2+^ release and propagation, and those which develop entirely non-spatial descriptions (Figure 8A). This section focuses primarily on theoretical and numerical approaches which enable large-scale tissue simulations. Analytical and statistical descriptions have also been presented but are not described in detail here; the reader is referred to Rovetti et al. (2007) and Asfaw et al. (2013), for example, where a mean first-passage-time approach was used to demonstrate how Ca^2+^-release depends on local properties within microdomains and to quantify measures of how events synchronize in tissue.

### Spatial Simplified Models

Williams et al. (2007, 2008) pioneered methods for efficient simulation of stochastic Ca^2+^ dynamics while preserving the importance of local control and features such as graded release. The first study (Williams et al., 2007) developed a probability density approach for modeling local control of CICR and compared results to a Monte-Carlo simulation regarding both validation and computational efficiency. As a simplification based on the “all-or-nothing” response of RyRs within a CRU, the RyRs in each CRU were described as a single “megachannel” i.e., treating them as a single channel which can only occupy a single state at once. Describing this megachannel with a simple two-state model enabled combination with a two-state model of the LTCCs to develop a four-state, minimal model of the state of the CRU. The probability density approach was then applied in the limit that the number of CRUs in the myocyte is sufficiently large (>5,000, which is less than the ∼20,000 observed in cardiomyocytes) by describing the probability that one would find a randomly sampled CRU in a specific state (S), associated with local dyadic Ca^2+^ (Ca_*ds*_) and Ca_SR_ concentrations:


(42) $$\rho^{i}\left(Ca_{ds},Ca_{SR},t\right)dCa_{ds}dCa_{SR}=\begin{array}{c} \mathrm{Pr}\{Ca_{ds}<\tilde{C}a_{ds}\left(t\right)<Ca_{ds}+dCa_{ds}\text{and}\\ Ca_{SR}<\tilde{C}a_{SR}\left(t\right)<Ca_{SR}+dCa_{SR}\text{and}\\ \tilde{S}\left(t\right)=i\} \end{array}$$

Where *i* is an index that runs over the four CRU states and tildes indicate random quantities. This must satisfy advection-reaction equations in order to make it equivalent to the Monte-Carlo approach. This approximation was combined with a deterministic solution to the bulk concentrations and a high resolution FDM approximation of spatial dynamics. The authors also derived a univariate approximation to the model based on the marginal density of Ca_SR_ jointly distributed with the CRU state, although the reader is referred to the original publication for further details beyond the scope of this overview. Importantly, they demonstrated that the approximation agreed strongly with the Monte-Carlo approach, which converges to this result so long as it contains a realistically large number of CRUs. The resulting method is ∼500 times more computationally efficient than the Monte-Carlo approach.

Subsequently, the approach was generalized to describe situations where the dynamics of Ca_*ds*_ are much faster than Ca_SR_ (Williams et al., 2008). The minimal four-state model of the CRU (two states for the RyR megachannel and two for the LTCCs) was expanded to a 12-state model, by extending the two-state RyR model to a six-state model that includes highly cooperative opening and an SR-Ca^2+^ dependence such that depletion of the SR-Ca^2+^ reduces the open probability. The model builds on the univariate approximation presented in the previous study, employing a moment-closure approach truncated at the second-order. The resulting method similarly agrees with the Monte-Carlo approach but with a substantial increase in computational efficiency by a factor of ∼10,000.

Chen et al. (2011) developed a simplified spatial model by imposing a number of reductions to a full description of spatial Ca^2+^ handling. The following two-assumptions drove model development: (1) Whereas Ca^2+^-waves nucleate at some location within the cell and propagate rapidly in the transverse direction, the greater length of the cell compared to its width implies that most Ca^2+^-wave propagation is approximately planar in the longitudinal direction; and (2) in general during a Ca^2+^-wave, all CRUs within a sarcomere are activated at approximately the same time. These assumptions enable a 1D lattice model to be constructed where each node represents a sarcomere (Figure 8A). The state of each sarcomere can be simplified into either being in a non-spark or spark condition [represented by 0 and 1 respectively and conceptually similar to the use of a single RyR megachannel as described in Williams et al. (2007, 2008) above]. The rate of spark recruitment was defined by:


(43) $$R\left(t\right)=R_{ICaL}\left(t\right)+R_{SCRE}\left(t\right)$$

Where *R*_*ICaL*_(t) is the recruitment rate due to activation by *I*_CaL_, and *R*_*SCRE*_(t) is the rate of recruitment via spontaneous sparks and waves. Assuming the release flux (*J*_rel_) associated with each spark is approximately exponential, the spark-rate, *R*(t), can be associated with *J*_rel_ via the following differential equation:


(44) $$\frac{dJ_{rel}\left(t\right)}{dt}=g\cdot Ca_{SR}\left(t\right)\cdot R\left(t\right)-\frac{J_{rel}\left(t\right)\cdot \left(1-\tau_{d}\left[dCa_{SR}/dt\right]/Ca_{SR}\right)}{\tau_{d}}$$

Where τ_*d*_ is the time-constant of the exponential function assumed to describe the spark flux. The rates of spontaneous spark initiation and a transmission-time and -probability [i.e., the components of *R*_*SCRE*_(t)] are primarily regulated by Ca_SR_ and were fit to experimental data to describe Ca^2+^-wave velocity, enabling many features of whole-cell SCRE to be captured at a significantly reduced cost.

In Hernandez-Hernandez et al. (2015) a similar model was constructed in 2D (Figure 8A). The state of each CRU (0 or 1 for non-spark or spark, respectively) was described by the following simple reaction:


(45) $$0\overset{\alpha =f\left(Ca_{i}\right)}{\underset{\beta }{⇌}}1$$

Inter-CRU coupling was described using a spatially exponential function such that the influence of one CRU on its neighbors decays rapidly as distance increases:


(46) $$h_{ij}=r_{j}e^{-{\left|x_{i}-x_{j}\right|}^{2}/l^{2}}$$

Where *r*_*j*_ is the Ca^2+^ released at site *j*, *x*_i_, and *x*_*j*_ are the locations of the two coupled CRUs and *l* is the diffusive length-scale. This model was used to evaluate the influence of CRU connectivity on Ca^2+^-wave nucleation and propagation.

In Romero et al. (2019) a 2D model was developed where the dynamics of each CRU were described by a non-linear map which relates Ca^2+^ concentrations from one beat to the next (rather than solving concentrations on a small time-step within each beat). This is a spatial analog of the iterative-map approach described in the next sub-section. Concentrations for beat *n*+1 are functions of concentrations and fluxes during beat *n*:


(47) $$Ca_{SR}^{x}\left(n+1\right)=Ca_{SR}^{x}\left(n\right)-R^{x}\left(n\right)+U^{x}\left(n\right)$$

(48) $$Ca_{i}^{x}\left(n+1\right)=Ca_{i}^{x}\left(n\right)+R^{x}\left(n\right)-U^{x}\left(n\right)$$

Where Ca_SR_*^x^* and Ca_i_*^x^* are the SR and intracellular Ca^2+^ concentrations at the spatial point *x* (=i,j in 2D), *R^x^*(n) is the total Ca^2+^ released from the SR at point *x* during beat *n* and *U^x^*(n) is the total Ca^2+^ pumped back into the SR at point *x* during beat *n*. As a simplification, it is assumed that the total Ca^2+^ is conserved (Ca_SR_ + Ca_i_ = C) and can be thusly normalized to 1 (arbitrary units). The stochastic dependence of intracellular Ca^2+^ release (*R^x^*) on voltage can be incorporated by accounting for the probability of release, and intracellular uptake is given as a function of the CaT peak. Ca^2+^ diffusion was then described by accounting for the average Ca^2+^ over the nearest neighbors in the 2D lattice of CRUs.

In Cantalapiedra et al. (2017) a spatial model was developed which included simplified descriptions of the LTCCs and RyRs through exploiting symmetries and other factors to substantially reduce the number of equations/parameters governing these dynamics; spatial coupling was described explicitly as in a CRU-grid fully spatial model. The model was used to study the influence of SR-Ca^2+^ load and RyR refractoriness on the dynamics of CaT alternans.

### Non-spatial Simplified Models

Further simplification can be achieved by developing non-spatial (or “0D”) approximations, which have no explicit description of spatial diffusion in the sub-cellular volume and present the opportunity for the largest increases in computational efficiency. Several different techniques have been proposed.

#### Reduction to (Semi-)deterministic Models

In Chen et al. (2012) a model was developed which describes single-cell SCRE dynamics using a single simple two-state reaction scheme that is able to capture the statistics of SCRE timing and magnitude. This is the same basic scheme as presented by Hernandez-Hernandez et al. (2015) and equation (45), but is now solved in the deterministic limit describing the whole-cell (Figure 8A). This was applied in a 1D model of tissue to analyze the relationship between the statistics of single-cell SCRE (pertaining to timing, magnitude and duration) and the emergence of spontaneous focal (or ectopic) excitation in tissue.

In studies by Shiferaw et al. (2018, 2020) a phenomenological, population-dynamics-like model of spark recruitment was developed that matched behavior of the 3D cell model. Here, rather than modeling CRU dynamics explicitly, the number of active CRUs (or number of sparks) were tracked and dynamically evolves dependent on the number of sparks initiated (Δ*n*^+^) and extinguished (Δ*n*^–^) at each time-step (Δ*t*):


(49) $$n_{i}\left(t+△t\right)=n_{i}\left(t\right)+△n_{i}^{+}-△n_{i}^{-}$$

The number of sparks initiated was determined by the rate at which sparks are recruited (either spontaneous or triggered) and stochasticity was maintained through the use of random number sampling. This used a similar approach to individual CRU recruitment to that of Chen et al. (2012) and Hernandez-Hernandez et al. (2015) but now included distinction between junctional and non-junctional sparks [both described by equation (45) but with different values for the transition rates], enabling the impact of heterogeneous and variable atrial T-systems to be captured in this reduced model. The approximation could reproduce both CaT alternans and SCRE. The model was used to study synchronization of SCRE in atrial tissue, developing focal excitations, conduction-block, and non-stable re-entrant-like excitation patterns.

#### Iterated-Map Models

Qu et al. (2007) developed an iterated-map model of CRU activity, relating Ca^2+^ and voltage properties on a beat-to-beat basis (Figure 8A), a whole-cell equivalent to the more recent approach presented in Romero et al. (2019). The approach can be briefly summarized by the following equations. Firstly, the APD can depend on both the diastolic interval (DI) and peak of the CaT (Ca*^peak^*). Thus, the APD at the current cycle (n) depends on the previous cycle (n-1) and is given by:


(50) $$APD_{n}=f_{1}\left(DI_{n-1}\right)+f_{2}\left(Ca_{n}^{peak}\right)APD_{n}$$

Where the functions of DI and intracellular Ca^2+^ have been separated: *f*_1_ is the APD restitution function and *f*_2_ accounts for the coupling strength between Ca^2+^ and APD (which can be positive or negative). The peak Ca^2+^ concentration at cycle *n* can be given by the sum of the diastolic Ca^2+^ from the previous cycle (*Ca*_*n*–1_) and the total Ca^2+^ released from the SR in the current cycle (*r*_*n*_):


(51) $$Ca_{n}^{peak}=Ca_{n-1}+r_{n}$$

The total Ca^2+^ released from the SR, *r*_*n*_, is given by:


(52) $$r_{n}=f_{3}\left(DI_{n-1}\right)f_{4}\left(CaSR_{n-1}^{load}\right)$$

Where *f*_3_ describes the restitution properties of SR-Ca^2+^ release (e.g., RyR refractoriness) and *f*_4_ describes the dependence of Ca^2+^ release on the SR-Ca^2+^ load. The reader is referred to the original publication for full details on the parameters and functions involved. This model reproduced the non-linear dynamics of Ca^2+^ handling including CaT alternans, and this simplification helped to develop a unified theory of CaT alternans in cardiac cells (Qu et al., 2016). Furthermore, this vast simplification in both space and time produces exceptionally efficient simulations, computationally less intense than standard, non-spatial common-pool models of cardiac cellular electrophysiology.

#### Models of Imposed, Stochastic Spontaneous Ca^2+^ Release Functions

As an alternative to the above approaches, it is also possible to control spontaneous CaTs in otherwise deterministic cell models by imposing (or clamping) SCRE waveforms (Figure 8A). In Xie et al. (2010), the intracellular release flux (*J*_rel_) associated with SCRE was controlled by imposing a waveform defined by two sigmoidal functions, the parameters of which determined the timing, duration and magnitude of SCRE:


(53) $$\begin{array}{c} J_{rel}^{spont}=G_{spont}{\left(1+e^{-\left(t-t_{0}\right)/\tau_{1}}\right)}^{-1}\\ {\left(1+e^{\left(t-t_{0}\right)/\tau_{2}}\right)}^{-1}\left(\frac{v_{ds}}{v_{jsr}}Ca_{SR}-Ca_{ds}\right) \end{array}$$

Where *G*_*spont*_ is a rate constant (set to 0.0674 ms^–1^ in the original study), *t*_0_ was set to 425 ms and τ_1_ = 10 ms and τ_2_ = 30 ms. The model was used to determine the minimum number of cells undergoing DADs in various tissue conditions in order for this to manifest as a focal excitation. Whereas not performed in the original study, the timing and duration parameters (*t*_0,_ τ_1_, τ_2_) could be randomly sampled from distributions to reproduce stochasticity in independent cellular SCRE. This approach was used in Liu et al. (2015), sampling from Gaussian distributions, in order to study the dynamics of independently timed DADs in tissue, revealing mechanisms of synchronization into focal excitation and DAD-mediated conduction block (e.g., Figure 8B). Liu et al. (2016) and Ko et al. (2017) used a similar but further simplified approach in which the Ca_i_ associated with SCRE was directly controlled, described by a Gaussian-shaped function with equivalent parameters to control timing, magnitude and duration:


(54) $$Ca_{i}^{spont}=Ae^{-{\left(t-t_{0}\right)}^{2}/2\sigma^{2}}$$

Where *A* sets the maximum amplitude, *t*_0_ sets the latency (timing) and σ sets the duration. In Colman et al. (2017a) and Colman (2019) an approach was developed which used sigmoidal functions to control the RyR-state directly, reproducing different shapes of long- and short-release waveforms. For short (spike-like) waveforms:


(55) $$N_{RyR\_O}=N_{RyR\_O}^{peak}{\left(1+e^{-\left(t-t_{1}\right)/k_{1}}\right)}^{-1}{\left(1+e^{\left(t-t_{2}\right)/k_{2}}\right)}^{-1}$$

(56) $$t_{1}=t_{i}+0.5\left(t_{p}-t_{i}\right)$$

(57) $$t_{2}=t_{p}+0.5\left(t_{f}-t_{p}\right)$$

(58) $$k_{1}=0.1689\left(t_{p}-t_{i}\right)+0.00255$$

(59) $$k_{2}=0.1689\left(t_{f}-t_{p}\right)+0.00255$$

where *t*_i_ is the initiation time (equivalent to latency, *t*_0_, in the above models) of the SCRE, *t*_f_ is the end time (duration, λ, thus = *t*_f_-*t*_i_), *t*_p_ is the time of the peak of the waveform and *N*_RyR_O_^peak^ is the peak proportion of open RyRs. In this model, *N*_RyR_O_ [equation (55)] replaces the “O” in the *J*_rel_ equation [equation (5)] and so *J*_rel_ and the CaT are allowed to dynamically evolve according to their deterministic functions; the magnitude of the CaT associated with the SCRE is therefore dependent on *N*_RyR_O_*^peak^*, the SR-Ca^2+^ and *J*_rel_ maximal flux rate. This model demonstrated feedback between re-entry and focal excitation in which the rapid activation during re-entry loads the SR-Ca^2+^ to promote focal excitations following re-entry termination (Figure 8B).

In all implementations, the parameters defining the distributions which describe SCRE statistics could be set as a function of environmental variables, such as SR-Ca^2+^, enabling the simplified cell model to respond to pacing with variable SCRE statistics in congruence with the dynamics of spatial Ca^2+^ cell models. All of these models employ an algorithm to determine if Ca_i_, *J*_rel_ or RyR-state is controlled by the deterministic cell model or undergoes the imposed SCRE clamped waveform, enabling integration with dynamically evolving deterministic cell models in both single-cell and tissue simulations. The computational efficiency of these models is comparable to that of standard common-pool models of cardiac electrophysiology; the largest computational cost is the generation of random numbers (where one must indeed be careful with implementations of parallelization in tissue simulations), but the cost of this inclusion is smaller than the typical differences in computational efficiency between common-pool models which feature a different number of components and governing equations.

### Estimating Probabilities of Rare Events

Walker et al. (2017) implemented a study in which 3D cell models were coupled in a 1D tissue strand (or fiber). They investigated the mediators of Ca^2+^ waves and DADs in single cells, and used the 1D fiber model to translate these features to tissue activity. From this, they developed a spatial-average filtering model which aimed to estimate *V*_m_ from intracellular release fluxes, enabling the estimation of the probabilities of “extreme” (i.e., rare) events in which multiple cells synchronously undergo large-scale SCRE, i.e., the requirements for focal excitations. This type of approach is powerful and important because the generation of a serious arrhythmia in an individual is often a very rare event that cannot be robustly or consistently captured in simulations of a generally normally functioning heart. In agreement with other studies (Liu et al., 2016; Campos et al., 2017; Colman, 2019), they found reduced *I*_K1_ and inter-cellular coupling to be important for enabling SCRE to overcome electrotonic load and promote focal excitations.

### Whole-Heart Models; Integration With Tissue Imaging

A few studies have integrated these reduced models of stochastic sub-cellular Ca^2+^ handling into models of the whole atria or ventricle in order to study the interaction between cellular function and tissue structure in controlling the emergence and dynamics of arrhythmogenic triggers. This is perhaps taken the furthest in a series of papers by Campos et al. (2015, 2017, 2018, 2019). The initial study (Campos et al., 2015) combined the phenomenological model of SCRE as proposed in Chen et al. (2011) with a full bi-ventricular 3D model which included a description of the Purkinje network. This model demonstrated that focal excitations were preferentially located to the Purkinje network due to the reduced electrotonic load in these pseudo-1D-strands, with focal excitations increasingly repressed as dimensionality increased from 1D to 3D (Figure 8C). Campos et al. (2017) subsequently incorporated a description of sodium-channel dysfunction, which promoted focal excitations and conduction block leading to re-entry. The two more recent studies (Campos et al., 2018, 2019) now combined these analyses with structural remodeling associated with infarcts in both idealized 2D sheets (Figure 8C) and realistic geometries in the 3D bi-ventricular model, demonstrating that the macroscopic and microscopic anatomy of the infarct region could promote both focal excitation and re-entry and highlighting the mechanisms by which fibrosis could increase the probability of focal excitations in these conditions.

These studies demonstrate structural features which can co-localize both focal excitation and re-entrant excitation. In Colman (2019) a purely functional mechanism which can co-locate focal and re-entrant excitation was revealed: the in-excited core of re-entrant excitation lead to substantially longer latency times in this region, enabling focal excitation to preferentially emerge from this same location (Figure 8B); interaction of focal excitation with the tail of the previous re-entrant excitation could lead to highly asymmetric focal excitations which themselves may degenerate back into re-entry with a core in the same approximate location. Thus, these studies are revealing both structural and functional mechanisms which spatially relate focal and re-entrant excitation.

### Drawing on Physical Analogies for Mechanistic Explanation

Other works have used detailed or simplified models to make analogies that relate cardiac Ca^2+^-handling phenomena to other physical phenomena, which may offer further insight into fundamental underlying mechanisms or provide more predictive power.

Alvarez-Lacalle et al. (2015) used both a 2D sub-cellular spatial model and simplified descriptions in order to analyze the dynamics of CaT alternans. Through scaling analysis of correlations near the transition to alternans it was demonstrated that CaT alternans could be described as an order-disorder phase transition, leading to an analogy to the Ising model of ferromagnetism in statistical mechanics. This was further generalized to describe more features of Ca^2+^ dynamics, including coupling with voltage, in Romero et al. (2019), where the analogy was extended to the more general Potts model. Both discontinuous first-order phase transitions and second-order continuous phase transitions (alternans) were observed to emerge under different conditions in cardiac cellular dynamics, further supporting the idea that statistical mechanics tools may be valuable for understanding cardiac function.

Conesa et al. (2020) presented a novel approach to understanding steady-state activity through analysis of a single beat which is not in homeostatic balance, by reduction to two-variable general equilibrium conditions in analogy to models of macro-economics. Such an approach can help to explain the complex and often counter-intuitive features of Ca^2+^-handling and offers substantial predictive power without the requirement for computer-intensive simulations.

## Summary and Conclusion

Recent advances in the robustness, complexity and sophistication of computational models of spatial Ca^2+^-handling from the nanometer-scale to the whole-heart scale are enabling advanced simulations to be performed to reveal fundamental properties of cardiac ECC in both health and disease. Multiple different approaches have been explored to describe nanodomain dynamics, model inter-CRU coupling, implement experimental imaging data, and translate models to the whole-heart scale. There are fundamental differences in the approaches and structure of these models, and in some cases model behavior can differ substantially (e.g., in triggered Ca^2+^ wave propagation). Nevertheless, the general agreement between models is encouraging and the availability of multiple different models provides the opportunity to comprehensively test hypotheses and explore fundamental theories. As computational power increases, experimental imaging data improves, and more powerful coarse-graining techniques are developed, the relevance, scope and power of these models will only continue to increase.

There are a number of challenges and avenues for future development and innovation. Robust validation of the models remains a major difficulty, not-least because of the challenges in obtaining sufficient and congruent experimental data to validate the many interacting model components and emergent functional phenomena. Validation of the governing RyR models themselves, in isolation and in the context of the spatial-nanodomain, is non-trivial - important features of Ca^2+^ sparks, such as the spatial FWHM, do not necessarily match experimental observations. This is not the only difficulty associated with obtaining a realistic and well-validated description of RyR gating: Integration of spark properties in the context of whole-cell homeostasis is also highly challenging due to the interaction with the wider model system and further constraints on model stability and long-term dynamics.

At the whole-cell scale, many approaches have been implemented to describe spatial Ca^2+^ coupling, corresponding to fundamentally different underlying model structures. Each approach has associated features of the CaT and Ca^2+^-handling dynamics, such as the robustness of Ca^2+^-wave propagation; certainly, not all of these structures and parameter combinations can simultaneously accurately describe real myocytes, and there are therefore fundamental questions about the mechanisms of inter-CRU Ca^2+^ diffusion which remain to be resolved. Nevertheless, the success of these models to explain and provide interpretations into experimental results is highly encouraging, especially in the more recent studies which combine experiment and simulation and are indeed beginning to resolve some of these issues.

One of the most exciting and challenging prospects is true multi-scale model integration. The recent spatial models of single nanodomains highlight the importance of specific RyR arrangement and microstructure of the dyad; incorporating these features into a whole-cell model containing tens of thousands of heterogeneous dyads is far from trivial. The detailed models of the local regions of a single T-tubule reveal important features of local Ca^2+^ gradients and channel distribution – combining these models with detailed representations of RyR arrangement in nanodomains, models of counter-ion fluxes, and the electrophysiology of the T-tubule membrane itself presents exciting prospects for powerful and highly accurate models of local control; again, translating these to the whole-cell scale is associated with a number of challenges. Initial success has been achieved in models attempting to preserve the impact of stochastic spatial Ca^2+^ dynamics in reduced, computationally efficient cell models suitable for tissue simulations, revealing the mechanisms of ectopic excitation, its interaction with re-entry, and dependence on tissue structure. Generalizing these models to naturally capture the dynamics of heterogenous populations of cells, themselves depending on heterogeneous sub-cellular structure, remains a major goal.

Finally, there have been substantial advances in approaches for image-based modeling, both for high-throughput, population-cohort simulations, and for direct integration of experimental structures. Closely related to advances in experimental imaging modalities, this is occurring at multiple spatial scales including the nanodomain, localized sub-cellular regions, whole-cells, and whole-heart. The further development and automation of these experimental-simulation frameworks presents exciting prospects for the true mechanistic analysis of structure-function relationships underlying cardiac electrophysiology from the nanometer to the whole-heart.

## Author Contributions

MC conceived the review, drafted and edited the manuscript, and prepared the illustrations. All the authors drafted and edited the manuscript.

## Conflict of Interest

The authors declare that the research was conducted in the absence of any commercial or financial relationships that could be construed as a potential conflict of interest.

## Publisher’s Note

All claims expressed in this article are solely those of the authors and do not necessarily represent those of their affiliated organizations, or those of the publisher, the editors and the reviewers. Any product that may be evaluated in this article, or claim that may be made by its manufacturer, is not guaranteed or endorsed by the publisher.
